# Changes in the Metagenome-Encoded CAZymes of the Rumen Microbiome Are Linked to Feed-Induced Reductions in Methane Emission From Holstein Cows

**DOI:** 10.3389/fmicb.2022.855590

**Published:** 2022-05-20

**Authors:** Kristian Barrett, Lene Lange, Christian F. Børsting, Dana W. Olijhoek, Peter Lund, Anne S. Meyer

**Affiliations:** ^1^Protein Chemistry and Enzyme Technology Section, DTU Bioengineering, Department of Biotechnology and Biomedicine, Technical University of Denmark, Lyngby, Denmark; ^2^LLa-BioEconomy, Research & Advisory, Valby, Denmark; ^3^Department of Animal Science, AU Foulum, Aarhus University, Tjele, Denmark

**Keywords:** Holstein cattle, microbiome, CAZymes diversity, cnserved unique peptide patterns (CUPP), methane emission

## Abstract

Enteric methane (CH_4_) emission from cattle is strongly linked to the feeding regime and the rumen microbial community structure. Here, we report that feed-induced CH_4_-reducing effects correlate with specific alterations in the profile of the microbiome-encoded carbohydrate-active enzymes predicted from the rumen fluid metagenome. Rumen microbiome samples were obtained by mouth-tube sampling from 12 lactating Holstein cows after 3–4 weeks of feeding with three different concentrate-to-forage-ratio diets, i.e., standard, high, and extremely high levels of concentrate (4 cows per group; constant dry matter intake in the three groups). Increased inclusion of concentrate involved increased starch levels in the diet at the expense of fiber. The extreme diet resulted in 48% reduction of the CH_4_ emission per kg dry matter intake compared to the standard diet. From metagenome sequencing of the rumen fluid samples from each cow, 561 different microbial strains (bins) could be derived from analysis of 260 billion DNA base pairs. In the cows fed, the extreme diet, the relative abundance of the majority of the bins, was significantly altered compared to the other groups. Fibrobacterota and Verrucomicrobiota were less abundant in the Extreme group. Surprisingly, no significant abundance changes were observed among Archaea and Bacteroidota, although abundance changes of individual bins of these phyla were found. For each of the 561 bins, the functions of the metagenome-encoded carbohydrate-active enzymes were predicted by bioinformatics using conserved unique peptide pattern (CUPP) analysis. By linking each of the predicted molecular functions of the enzymes to their substrates, changes were found in the predicted abundance of the different enzyme types. Notably, the decreased CH_4_ emission of the extreme diet group was concurrent with a profound decrease in the xylan-active enzymes, targeting the xylan backbone β-1,4-linkages, acetyl-, feruloyl-, and methyl-glucuronoyl substitutions in xylan. This work provides a first enzyme-conversion-based characterization of how extreme feeding, i.e., lowered forage, can drive rumen microbiome changes that support decreased CH_4_ emission *via* a changed carbohydrate-active enzyme profile. The data, furthermore, provide a metagenome-wide catalog of enzymes, underpinning the microbial conversion of different feed fibers (the enzymes attacking specific carbohydrate linkages) in the rumen of Holstein cows.

## Introduction

Emission of enteric methane (CH_4_) from cattle is getting worldwide attention due the increased awareness of its significant contribution to climate change. In Denmark, for example, enteric CH_4_ currently constitutes 36% of total agricultural greenhouse gas emissions (Albrektsen et al., 2017). The producers of CH_4_ in the cow rumen are the archaeal methanogens, converting primarily carbon dioxide, methanol or other C1 compounds, together with hydrogen (H_2_), during rumen fermentation into CH_4_.

One approach to reduce CH_4_ is through alteration of the diet and, thereby, rumen fermentation (Aguerre et al., 2011). For ruminants, forage traditionally constitutes a major part of the feed. When the ratio of forage to concentrate is increased, it leads to an increase in enteric CH_4_ emissions in ruminants (Aguerre et al., 2011; Rønn et al., 2022b), including Holstein cattle (Aguerre et al., 2011; Rønn et al., 2022c). The increased CH_4_ production is due to an increase in production of acetate and butyrate at the expense of propionate, thereby making more hydrogen available for methanogenesis.

The dominant phyla in the rumen of Holstein cattle include Bacteroidota, constituting about half, and Firmicutes, accounting for about a third of the total. Archaea are reported to constitute only a small quantity, equivalent to 0.5–1% of the total DNA in the rumen microbiome of Holstein cattle (Bharanidharan et al., 2021). Carbohydrate processing enzymes (CAZymes) are classified into families (CAZy families) based on their amino acid sequence similarity (Lombard et al., 2014; Drula et al., 2021). Archaea are notoriously known to encode few CAZymes, whereas bacterial species belonging to, e.g., Bacteroidota and Firmicutes are endowed with a strong palette of various CAZymes.

Efficient digestion of fibrous feeds has, for a long time, been crucial for increasing ruminant performance. It has been suggested that substituted hemicellulose, inherently including, e.g., acetylations of xylan fibers, could be a limiting factor in degradation of fibers in the rumen, indicating enzymatic modification to be the key for uptake (Titgemeyer et al., 1991). Major changes have recently been reported in the content of CAZymes upon altering the forage-to-concentrate ratio in the feed for Holstein cattle (Wang et al., 2019; Olijhoek et al., 2022). For example, it was found that cows fed high-forage feed, i.e., standard feed; the microbial diversity of CAZyme producers was high, and the abundance of certain glycoside hydrolases (GH), e.g., family GH 3 enzymes, was particularly high compared to cows fed a low-forage diet (Wang et al., 2019).

Although the catalytic mechanism is, in general, the same among enzymes belonging to the same CAZy family, the detailed substrate specificity can vary, which is why a deeper sub-grouping can help predict the enzyme function (specificity). One way to subgroup CAZymes is by categorizing them according to similarity of conserved peptide motifs in each protein *via* the CUPP algorithm (CUPP: conserved unique peptide patterns; Barrett and Lange, 2019; Barrett et al., 2020a). Recently, sub-grouping of enzymes within protein families or subfamilies by means of the CUPP algorithm has thus been shown to be congruent with a functional grouping of CAZymes (Barrett et al., 2020a).

The current study was undertaken to assess whether a correlation exists between the rumen metagenome-encoded CAZymes of Holstein cows and feed-induced changes in the cows’ enteric CH_4_ emission. The research strategy was thus designed to test if alterations in the Holstein cow rumen microbiome coincided with changes in the abundance and function of metagenome-encoded CAZymes active on the particular feed polysaccharides present. The rumen samples analyzed originates from a recent experiment with 12 lactating Holstein cows divided into 3 groups fed different diets, where a 48% reduction in CH_4_ was observed when a standard diet was replaced with a diet extremely high in concentrate and without grass and corn silage (Olijhoek et al., 2022). This high reduction in CH_4_ was accompanied by a decrease in the proportion of acetate to propionate from 2.70 in the standard diet to 1.55 in the group fed the diet, which was extremely high in concentrate; at present, we interpret this change as the extreme group being in a state of subacute acidosis. Our immediate explanation is, currently, that the metabolism in the Archaea is changed by the change in pH, which may cause a shift from hydrogenotrophicmethanogenesis toward a more acetoclasticmethanogenesis metabolism, thus allowing for H_2_ accumulation while leaving the abundance of Archaea constant (Wormald et al., 2020) – but the detailed metabolism is currently being investigated further in a separate study. The focus of the current work was to characterize changes in the rumen microbiome and assess if the prevalent CAZymes corresponded to feed-induced reduction in enteric methane emission in Holstein cows.

## Materials and Methods

The samples for the current work were obtained from a feeding study in cows that were fed for 3–4 weeks with specified diets, followed by 3-day stay in a respiration chamber (Olijhoek et al., 2022); the feeding study included 12 Holstein dairy cows that were randomly divided into three groups of four cows each. Each group received a different diet (Supplementary Table 1): A standard diet rich in grass silage and corn silage (standard), a high-concentrate diet (high), and an extremely high-concentrate diet without grass silage and corn silage (extreme). Concentrate typically includes grains, oil seeds, high protein seeds like peas and beans, and any byproducts from these sources. In broad terms, that leaves for forages: grasses, legumes, whole crops of cereals as maize, wheat, barley, etc. For the three groups, the average dry matter intake (DMI) per cow was 22.4, 23.6, and 22.1 kg per day for the Standard, High, and Extreme groups, respectively, but daily CH_4_ production, and thus the daily CH_4_ L/kg DMI, was significantly higher for cows fed the standard diet as compared to cows on the extreme diet (Table 1). Gas exchange (methane, carbon dioxide, oxygen, and hydrogen) was measured on individual cows in open circuit respiration chambers as explained in Olijhoek et al. (2022) using indirect calorimetry according to Hellwing et al. (2012). The cows were confined to the chambers throughout the measurement period with an airflow rate of 2,000 L/min.

**TABLE 1:** Data from *in vivo* measurements of dry matter intake (DMI), energy-corrected milk (ECM) yield, CH_4_, and H_2_ as modified from Olijhoek et al. (2022).

Diet	Intake and milk yield		Gas emission			
	DMI, kg/day	ECM, kg/day	CH_4_, L/day	CH_4_, L/kg DMI	H_2_, L/day	H_2_, L/kg DMI
Standard	22.4^A^	34.2^A^	561^A^	25.3^A^	10.5^A^	0.47^B^
High	23.6^A^	36.2^A^	518^A^	22.0^A^	16.7^A^	0.70^A,B^
Extreme	22.1^A^	29.6^A^	295^B^	13.2^B^	26.3^A^	1.19^A^
SEM	0.92	2.7	20.6	1.01	2.99	0.12

*Gas emission from Holstein cows fed different diets; a standard diet rich in grass silage and corn silage (Standard), a high-concentrate diet (High), and an extremely high-concentrate diet without grass silage and corn silage (Extreme; Olijhoek et al., 2022)^1^. ^1^PROC MIXED in SAS (version 9.4) was applied to construct a modified model of the statistical model used in the larger feed experiment (Olijhoek et al., 2022). Different superscript letters indicate a significant difference between groups at p ≤ 0.05.*

Hydrogen (H_2_) emission per kg DMI was significantly higher in the Extreme group than in the Standard group, but energy-corrected milk (ECM) yields were not significantly affected by diet, although the cows on the Extreme diet had numerically lowest ECM yield (Table 1). (Oxygen and carbon dioxide exchange did not differ between diets (Olijhoek et al., 2022).

Monosaccharide composition analysis of the three diets was acquired from the extreme feeding experiment (Olijhoek et al., 2022), Table 2. The analysis was performed as described by Knudsen (1997) with the exception that it included 1 h hydrolysis with 2 M H_2_SO_4_ instead of 2 h with 1 M H_2_SO_4_ (Knudsen, 1997).

**TABLE 2:** Monosaccharide composition analysis of the three diets, including the content of Rha (rhamnose), Fuc (fucose), Ara (arabinose), Xyl (xylose), Man (mannose), Gal (galactose), Glc (glucose), and U.A. (uronic acid).

Diet	Polysaccharides excluding starch and cellulose (% of dry matter)							
	Rha	Fuc	Ara	Xyl	Man	Gal	Glc	U.A.
Standard	0.3	0.1	5.1	7.3	0.5	1.7	1.6	5.6
High	0.3	0.1	6	6.3	0.6	1.8	1.7	5.4
Extreme	0.3	0.1	7	5.5	0.6	1.8	1.9	6.2

### DNA Extraction

DNA extraction was performed using the standard protocol for FastDNA Spin kit for Soil (MP Biomedicals, United States), with the following exceptions: 500 μL of a sample, 480 μL a sodium phosphate buffer and 120 μL MT. Buffers were added to a Lysing Matrix E tube. Bead beating was done at 6 m/s for 4 s × 40 s (Albertsen et al., 2015). DNA size distribution was evaluated using gel electrophoresis on the Tapestation 2200 with Genomic DNA screentapes (Agilent, United States). DNA concentration was measured with the Qubit dsDNA HS Assay kit (Thermo Fisher Scientific, United States). The samples intended for Nanopore sequencing were further purified and size selected with a custom SPRI protocol. Briefly, a custom bead buffer (10-mM Tris-HCl, 1 mM pH 8. EDTA, 1.6 M NaCl, 11% PEG) with washed AMPureXP beads (Beckman Coulter, United States) was added to 3-μg DNA at a bead-sample ratio of 0.7x. The beads were subsequently magnetically separated from the liquid phase and washed two times with 80% ethanol. The DNA was eluted in 100 μL nuclease-free water (Qiagen, Germany). The DNA size distribution was evaluated on the Tapestation 2200 using Genomic DNA screentapes (Agilent, United States). The final DNA concentration and purity (A260/A280 and A260/A230) were measured with the Qubit dsDNA HS Assay kit (Thermo Fisher Scientific, United States) and the Nanodrop ND-ONE-W device (Thermo Fisher Scientific, United States).

### Illumina Sequencing (DNA Sequencing)

Sequencing libraries were prepared using the NEB Next Ultra II DNA library prep kit for Illumina (New England Biolabs, United States) according to the manufacturer’s protocol. The sequencing libraries were pooled in equimolar concentrations and diluted to 4 nM. The samples were paired-end sequenced (2 bp × 151 bp) on a HiSeq (Illumina, United States), following the standard guidelines for preparing and loading the samples on the HiSeq platform.

Nanopore sequencing and data preprocessing – Three long-read sequencing libraries were prepared according to the SQK-LSK109 protocol. Approximately, 50–75 fmole was loaded onto primed FLO-MIN106D (R9.4.1) flow cells with 1,272–1,568 available pores and sequenced in MinKnow Release 19.12. Fast5 files were basecalled in Guppy v. 4.0.11 using the high-accuracy (hac) algorithm. Basecalledfastq data were subsequently adapter trimmed in Porechop v. 0.2.4 Porechop using default settings (Wick, 2018). NanoPlot v.1.27 (Coster et al., 2018) was used to assess quality parameters of the basecalled data. The trimmed data were then filtered in Filtlong v. 0.2.0 with a min_length set to 1,000 bp and a min_mean_q set to 90.

Metagenome assembly and binning – The Illumina sequence reads were trimmed for adaptors using cutadaptv. 1.16 (Marcel, 2011). The holo-metagenome was initially assembled with flyev.2.7.1 (Kolmogorov et al., 2019) by setting the genome size -g parameter to 1,600 m and invoking the metagenome parameter –meta. The assembled metagenome was subsequently polished with filtered Oxford Nanopore data, using one round of polishing with minimap 2 v. 2.17-r941 (Li, 2018) and racon v.1.4.13 (Vaser et al., 2017) and two rounds of polishing with medakav.1.0.3. The metagenome was finally polished with minimap 2 v. 2.17-r941 (Li, 2018) and racon v.1.4.13 (Vaser et al., 2017) using Illumina data from all three samples. The reads were mapped back to the assembly using minimap 2 v. 2.17-r941 (Li, 2018) to generate coverage files for metagenomic binning. Genome binning was carried out using metabat 2 v. 2.12.1 (Kang et al., 2019). Bin completeness and contamination were assessed using CheckMv. 1.1.2 (Parks et al., 2015). Genome bins were classified using GTDB v. 1.1.1 (Parks et al., 2019), and rRNA sequences were extracted using barrnap v. 0.9, github.com/tseemann/barrnap. The bins were annotated using PROKKA v. 1.14.0 (Seemann, 2014). A bin is considered a partial or fully metagenome-assembled genome, having completeness of at least 10%. The average completeness for the included bins was 52.4%.

Annotation of carbohydrate-active enzymes – The resulting proteins of the metagenome assembly were annotated to CAZy families by the CAZy groups using a combination of methods, including an internal HMM model (Lombard et al., 2014). Even though the majority of the CAZy families are targeting specific substrate types, the target linkage in the substrate or the specificities is, in some cases, diverse. For this reason, we performed a more nuanced functional prediction of the CAZymes, involving further categorization based on resemblance of peptide patterns (Barrett and Lange, 2019) in an attempt to break down the families into groups of more homogenous functional groups. To get further into each of the individual families and what their likely molecular substrate-degrading function is, each of the CAZymes has been functionally assessed, and the likely polysaccharide target and the likely monosaccharide target with the polysaccharide have been determined. Furthermore, the molecular functions of each of the CAZymes were predicted by CUPP (Barrett and Lange, 2019) using the CUPP.INFO online server (Barrett et al., 2020a). The polysaccharide substrates of each of the molecular functions was assigned as described in the Supplementary Table 2. The CAZy family and linkage target abundancies were normalized to 100% per diet group before comparison.

The Separation score is the distance between the centroids of two clusters divided by the average distance from the centroid to the members of the cluster (equation below), where *m* is the first group and *n* is the second group, *c* is the centroid of the group, whereas *p* the individual points within a group:

$$f\,\left(x\right)=\frac{\left|c_{m}-c_{n}\right|}{\sum_{i=1}^{m}\left|p_{i}-c_{m}\right|+\sum_{j=1}^{n}|P_{j}-c_{n}}$$

Statistical analysis – The correlation between the 12 methane measurements for the cows and the 12 individual abundances of a given type of CAZymes (the linkage target) was done by Spearman rank correlation. The values were determined using the python module “scipy.stats.spearmanr” with default settings. To assess when the change between the Standard and the Extreme groups was significant, the python module “scipy.stats.ttest_ind” was used with default setting given the individual values for the cows for the two groups. When the *p*-value was ≤ 0.05, the abundance was considered significantly changed.

The MDS plots – The dendrogram was created based on an observation matrix where bins were present in the first dimension and the individual linkage targets were the second dimension with binary values (True if the linkage target was found in the bin and False if the linkage target was absent in the bin). The observation matrix was converted into a distance matrix through the python module “scipy.spatial.distance.pdist” using the binary “Jaccard” similarity score. This distance matrix was then converted into a dendrogram using the python module “scipy.cluster.hierarchy.linkage” using the metric “Ward.”

## Results

As mentioned above, the present research is a part of a larger animal experiment; the main study design and overall feed response are outlined in (Olijhoek et al., 2022).

### Taxonomic Analysis of the Metagenome Data

For the rumen samples of each cow, between 18 and 27, giga-base pairs (Gbp) were sequenced (after trimming of the reads) and assembled into the 561 bins considered in this analysis; this metagenome assembly resulted in about one Gbp of assembled DNA. The metagenomes of the 12 individual cows were assembled into 561 bins with completeness of at least 10%, and the taxonomical composition on a phyla level was assessed (Table 3). A key observation was that there was no change for the two archaeal phyla, Crenarchaeota and Euryarchaeota between any of the feed groups, although two of the nine. Methanobrevibacter_Aspp were significantly more abundant in the Standard diet group. Likewise, there were no changes in the Bacteroidota phylum – a phylum harboring more than 25% of the bins (Table 3). However, among the rest of the phyla, several major changes were evident. Remarkably, Actinobacteriota increased by more than 15-fold from Standard to Extreme, but there was only a negligible difference between Standard and High. Oppositely, the (single member of the) Verrucomicrobiota phylum essentially disappeared, and the Fibrobacter phylum was profoundly decreased in the Extreme diet group compared to the levels found in the Standard and the High groups. Generally, Firmicutes (including phyla Firmicutes, Firmicutes A and Firmicutes C) increased more than 2.5-fold from Standard to Extreme (Table 3). The abundance of the members in phylum Desulfobacterota_A was nearly absent in the Standard Diet group but increased by over 100-fold in the Extreme group. Overall, there were hardly any changes between the Standard and the High feeding groups when compared to the Extreme group. Only the Proteobacteria did not follow this trend, since the Standard group was the only group with low abundance as both High and Extreme were similar, both having an increased abundance level of Proteobacteria.

**TABLE 3:** The abundance change summaries of the 561 assembled genomes by the phylum of the individual bins (a bin indicates a full or partially assembled genome in the rumen microbiome; only the DNA, which could be assembled into bins with completeness of at least 10%, is included).

Phylum	Abundance				
	Standard	High	Extreme	Change from Standard to Extreme	No. of bins
Crenarchaeota (Archaea)	0.011 ± 0	0.016 ± 0.01	0.011 ± 0.01	(No change)	1
Euryarchaeota (Archaea)	0.28 ± 0.1	0.24 ± 0.05	0.34 ± 0.14	(No change)	13
Actinobacteriota	0.56 ± 0.3^A^	0.39 ± 0.21^A^	8.61 ± 3.63^B^	Increase	18
Bacteroidota	14.4 ± 1.97	19.3 ± 2.74	19.4 ± 4.39	(No change)	157
Chloroflexota	0.01 ± 0	0.009 ± 0	0.008 ± 0.01	(No change)	1
Cyanobacteria	0.012 ± 0.01	0.025 ± 0.02	0.31 ± 0.29	(No change)	5
Desulfobacterota_A	0.007 ± 0.01^A^	0.016 ± 0.01^A^	0.10 ± 0.01^B^	Increase	1
Fibrobacterota	0.079 ± 0.02^A^	0.073 ± 0.03^A^	0.012 ± 0.01^B^	Decrease	4
Firmicutes	0.44 ± 0.13^A^	0.46 ± 0.11^A^	4.28 ± 1.32^B^	Increase	30
Firmicutes_A	8.04 ± 2.03^A^	7.5 ± 0.65^A^	16.1 ± 5.46*^B^*	Increase	272
Firmicutes_C	0.41 ± 0.08^A^	0.62 ± 0.19^A^	2.34 ± 0.54^B^	Increase	11
Patescibacteria	0.058 ± 0.01	0.072 ± 0.03	0.151 ± 0.08	(No change)	14
Proteobacteria	2.44 ± 1.48^A^	7.25 ± 3.55^B^	7.72 ± 2.63^B^	Increase	4
Verrucomicrobiota	0.027 ± 0.01^A^	0.012 ± 0.01^A^	0 ± 0^B^	Decrease	1
Unknown phylum	0.20 ± 0.04	0.29 ± 0.14	0.13 ± 0.05	(No change)	29

*Groups having different superscript letters indicate a significant difference between them across the diets, p-value = 0.05. Total abundance has been normalized to 100% of the total DNA sequences.*

**FIGURE 1:**
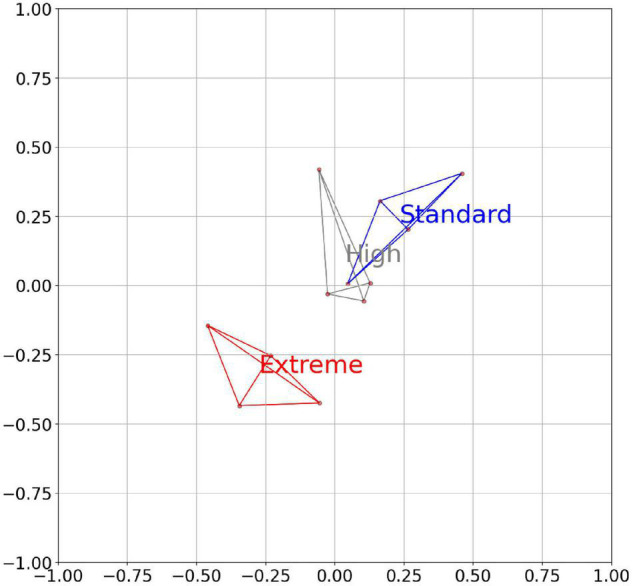
An MDS plot of the individual cows based in the abundance of the bins from the rumen. The clustering of results from the four cows of each feeding regime is indicated by color (blue, Standard; yellow, High; and red, Extreme). The axes are arbitrary values resulting from the reduction of the multidimensional space of correlation similarities into two dimensions.

When the metagenome data of 12 cows were analyzed based on the abundance changes of each of the bins individually, it was possible to observe clear separation of the cows from the Standard and the Extreme feeding groups. Based on the bin abundance change between the individual cows, clustering according to diet is observed (Figure 1). Notably, the data for the High Feeding group appeared relatively similar to those of the Standard group as Extreme separated considerably from Standard and High; while Standard and High overlapped. The separation score between the Standard and the Extreme was 1.56. In summary, the data infer that the feed changes induced a replacement of strains at the genus level to result in the emergence of a few dominant species.

When zooming in on the changes of the individual bins, a more nuanced picture emerged. Hence, profound differences in the abundance changes of the bins within each phylum were evident (Table 4). First, and most importantly, the individual bins within a given phylum in most cases did *not* follow the overall trend of the phylum, as the trend was often driven by a major change of the abundance of a few dominant bins within the phylum. The presence of a few dominant bins was most evident in the Extreme Feed group, where the diversity was, moreover, reduced compared to the Standard group. For example, for the Firmicutes_A, 124 bins were significantly more abundant in the Standard group (relative to the Extreme group), whereas, at the same time, 42 bins were significantly more abundant and dominant in the Extreme group (versus the Standard group). Hence, the majority of the bins within the Firmicutes_A phylum were most abundant in the Standard group, and thus shifted differently than what could be interpreted just based on the overall summarized abundance of the phylum, even though the total abundance of the bins in the Firmicutes_A phylum showed the opposite trend (Table 3). The exact same trend was evident for all Firmicutes phyla, i.e., including Firmicutes, Firmicutes_A, and Firmicutes_C (Tables 3, 4); the total abundance was greater for the Extreme group as compared to the Standard group, but the majority of the individual bins were more abundant in the Standard group and represented smaller abundance contributions from a higher number of bins (Table 4).

**TABLE 4:** Inspection of the abundance changes for the individual bins without each of the phyla.

Phylum	Total bins	Distribution of bin abundance		
		Standard	Extreme	No. of bins that are not significantly different among feed groups
Crenarchaeota (Archaea)	1	0	0	1
Euryarchaeota (Archaea)	13	2	0	11
Actinobacteriota	18	3	7	8
Bacteroidota	157	100	23	34
Chloroflexota	1	0	0	1
Cyanobacteria	5	1	0	4
Desulfobacterota_A	1	0	1	0
Fibrobacterota	4	3	0	1
Firmicutes	30	8	9	13
Firmicutes_A	272	124	42	106
Firmicutes_C	11	3	2	6
Patescibacteria	14	6	0	8
Proteobacteria	4	1	2	1
Verrucomicrobiota	1	1	0	0
Unknown phylum	29	22	3	4
**Total**	**561**	**274**	**89**	**198**

A second major point in the more nuanced picture was that some bins in a phylum did not follow the overall trend of that phylum. An example of this phenomenon was particularly evident for the Actinobacteriota. Specifically, three unknown species (two belonging to the genus UBA9715 and one to genus UBA1367) were thus found to be significantly more abundant in the Standard group as compared to the Extreme group, and, at the same time, seven species followed the common trend of the Actinobacteriota phylum (Table 3), i.e., their abundance was higher in the Extreme group compared to the Standard and High groups (Table 4). Specifically, these seven species included one species annotated to genus *UBA7741* (and named *UBA7741 sp900314495*), three species belonging to *Olsenella* (*Olsenella_Cumbonata*, *Olsenella sp900314535*, and *Olsenella_B* spp.), and three species from three different genera (*Olegusella sp900315165*, *RUG033 sp900314665*, and *QAMH01* spp.; Supplementary Table 3).

### The Metagenome-Encoded Carbohydrate Active Enzymes

To assess if the feed-induced changes in the microbiome might be related to the capability of the rumen microbes to degrade the particular carbohydrate sources in the new feed, the CAZymes encoded by the rumen metagenome were annotated for each of the bins, and the changes occurring between feed groups were examined. From the assembled DNA, above one million, exactly 1,050,464, encoded proteins were identified from gene prediction. Of those, a total of 17,461 proteins were annotated to contain at least one carbohydrate-active enzyme domain, and these were found to span over 91 different CAZy families, which are known to contain enzyme members involved in agricultural crop fiber degradation and polysaccharide modification.

**FIGURE 2:**
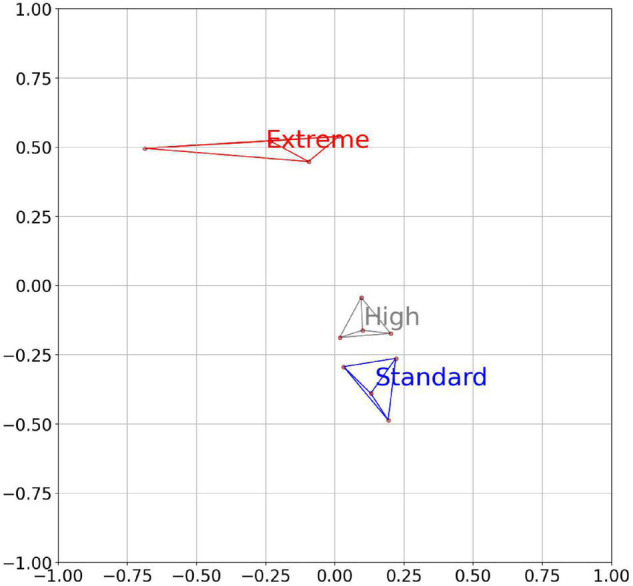
An MDS plot of the individual cows based on the abundance of the predicted CAZy families (91 different families) across all bins of the rumen metagenome samples. The colored clusters represent the members of the three different diet groups. The separation score between the Standard and the Extreme is 2.15. The axes are arbitrary values, resulting from the reduction of the multidimensional space of correlation similarities into two dimensions.

The abundance of the metagenome-encoded CAZymes belonging to the same family among the 91 CAZy families was summarized to determine abundance changes between the individual cows and the feed groups across all the 561 bins. Based on the abundance changes of the proteins in the 91 individual CAZy families, the cows in the different feed could be clustered in a pattern congruent with that of Figure 1 (Figure 2). In the clustering, based on CAZyme profiles, the cows belonging to the Extreme feeding group were clearly separated from the other two feeding groups.

From this assessment, the relative abundance of proteins belonging to 48 of these CAZy families was found to be significantly changed between the Standard and the Extreme Feed groups: 28 were relatively more abundant in the Extreme feeding group (CE4, GH1, GH2, GH3, GH13, GH27, GH30, GH32, GH53, GH57, GH59, GH63, GH76, GH81, GH88, GH93, GH98, GH106, GH119, GH128, GH133, GH143, GH146, GH148, PL4, PL9, PL27, and PL40), and 20 families were relatively more abundant in the Standard group (CE6, CE7, CE15, GH8, GH9, GH17, GH26, GH31, GH39, GH45, GH50, GH54, GH55, GH105, GH116, GH139, GH144, GH147, PL10, PL26, PL37, and PL38).

### Linking the CAZymes to Feed Substrates

To understand what the likely molecular function of each of the CAZymes in relation to substrate is, a further functional assessment was conducted to obtain a more detailed functional prediction, i.e., prediction of which bond the enzyme is attacking in the feed. This assessment resulted in 84 different substrate linkage targets, of which 28 were found to be significantly changed when the relative abundance was compared between the Standard (Table 5) and the Extreme groups (Table 6).

**TABLE 5:** Significantly changed substrate linkage targets between the Standard group and the Extreme group across all bins, which are most abundant in the Standard group.

Standard – Predicted polysaccharide targets of microbiome CAZymes			Relative abundance			Methane correlation	Included Families
			
Major polysaccharide	Polysaccharide branch	Specific target	Standard	High	Extreme		
Pectin	Arabinogalactan	α-1,3/α-1,5 :: Ara	10.6 ± 0.3	10.5 ± 0.2	8.4 ± 0.8	0.87	7
–	–	β-1,2 :: Ara	1.4 ± 0.1	1.5 ± 0.3	1 ± 0.2	0.76	3
–	Homogalacturonan	α-1,4 :: (D4)GalA	0.7 ± 0.1	0.6 ± 0.1	0.3 ± 0.1	0.87	4
–	–	Methylation	2.1 ± 0.1	2.1 ± 0.1	1.7 ± 0.1	0.72	1
–	–	α-1,4 :: Rha	1.7 ± 0.1	1.7 ± 0.2	1 ± 0.2	0.78	1
–	Rhamnogalacturan II	β-1,4 :: GlcA	1.5 ± 0.1	1.4 ± 0.1	1 ± 0.1	0.66	1
	–	β-1,2 :: Ara	0.9 ± 0.1	0.9 ± 0.1	0.6 ± 0.1	0.78	2
–	Xylogalacturonan	α-1,4 :: GalA{Xyl}	2 ± 0.2	2 ± 0.2	1.2 ± 0.2	0.85	1
Xylan	Glucuronoarabinoxylan	α-1,2 :: GlcA{4Me}	0.9 ± 0.1	0.9 ± 0	0.6 ± 0.1	0.86	2
–	–	Methylation	0.7 ± 0	0.6 ± 0.1	0.1 ± 0.1	0.85	1
–	Xylan – Backbone	Acetylation	2.8 ± 0.1	2.8 ± 0.3	1.5 ± 0.2	0.78	7
	–	Ferulic acid	1.1 ± 0	1 ± 0.1	0.7 ± 0.1	0.77	1
–	–	β-1,4 :: Xyl	6.2 ± 0.4	5.9 ± 0.8	3.7 ± 0.3	0.79	13
Xyloglucan	Xylose substitutions	α-linked Xyl	1.9 ± 0.1	1.7 ± 0.1	1.1 ± 0.2	0.88	1
Arabinogalactan proteins	Arabinogalactan	β-linked GlcA	0.8 ± 0	0.8 ± 0.1	0.7 ± 0.1	0.40	4
Pullulan	Pullulan – Branch	α-linked Glc	1.2 ± 0.1	1.3 ± 0.1	0.9 ± 0.2	0.71	1

*The functions of all the CAZy families add up to 84 substrate linkage targets, of which the 28 included had a significant change between the Standard and the Extreme groups (t-test with a p-value below 0.05 and least 20% change). The specific targets are shown as the type of bond attacked in a feed polysaccharide. The column showing methane correlation is a Spearman rank correlation between the abundance of the given linkage target and the methane emission from the cows.*

**TABLE 6:** Significantly changed linkage targets between the Standard group and the Extreme group across all bins, which are most abundant in the Extreme group.

Extreme – Predicted polysaccharide targets of microbiome CAZymes			Relative abundance			Methane correlation	Included families
			
Major polysaccharide	Polysaccharide branch	Specific target	Standard	High	Extreme		
Cellulose	Cellulose – Backbone	β-1,4 :: Glc	4.8 ± 0.6	4.4 ± 0.1	7.4 ± 0.2	–0.68	7
Xyloglucan	Fucose substitutions	α-linked Fuc	0.2 ± 0	0.2 ± 0	0.5 ± 0.1	–0.53	3
Glucan	Glucan – Backbone	β-1,3 :: [Glc]n	0.9 ± 0.1	1.1 ± 0.1	1.3 ± 0.2	–0.82	11
–	Undefined	Undefined	0.8 ± 0.1	0.9 ± 0.1	1.3 ± 0.2	–0.84	14
Inulin	Undefined	Undefined	1.1 ± 0.1	1.2 ± 0.2	1.6 ± 0.3	–0.78	2
Homoxylan	Homoxylan – Backbone	β-1,3 :: Xyl	0.9 ± 0.1	0.9 ± 0.1	1.3 ± 0.1	–0.83	1
Arabinogalactan proteins	Galactose substitutions	α-1,3 :: Gal	0.6 ± 0.1	0.7 ± 0.1	0.8 ± 0.1	–0.69	1
Starch	Starch – Attachments	α-linked Glc	2.3 ± 0.4	2 ± 0.1	3.7 ± 0.4	–0.64	1
–	Starch – Backbone	α-1,4 :: Glc	1.3 ± 0.3	1.3 ± 0.2	3 ± 0.9	–0.79	1
–	–	Phosphate	0.2 ± 0.1	0.2 ± 0.1	1 ± 0.3	–0.64	1
–	–	α-1,4 :: [Glc]n	1.4 ± 0.2	1.4 ± 0.2	3 ± 1.1	–0.76	3
Pectin	Arabinogalactan	Attached Ara	0.9 ± 0.1	0.9 ± 0.1	1.3 ± 0.1	–0.83	1
–	Undefined	Undefined	2.3 ± 0.1	2.3 ± 0.2	3.4 ± 0.2	–0.83	3

*The functions of all the CAZy families add up to 84 substrate linkage targets, of which the 28 included had a significant change between the Standard and the Extreme groups (t-test with a p-value below 0.05 and least 20% change). The column methane correlation is Spearman rank correlation between the abundance of the given linkage target and the methane emission from the cows.*

From Tables 5, 6, it is possible to discern a trend that the polysaccharide linkage targets, which are significantly more abundant in the Standard feed group, seems to be associated with pectin or xylan when the ratios of CAZmes are compared. Particularly, methylated homogalacturonan and acetylated β-,4-xylan and methyl-glucuronoxylan linkage targets were relatively more prevalent in the Standard feed group compared to the Extreme group (Table 5). On the other hand, the microbiome of the Extreme feeding group was predicted to encode for relatively more enzymes targeting cellulose, β-1,3glucan, and phosphorylated starch (Table 6). Additionally, enzymes targeting α-1,3 linked galactose in arabinogalactan protein and backbone β-1,3 homoxylan linkages were similarly more abundant in the Extreme feed group when the ratios were compared.

**FIGURE 3:**
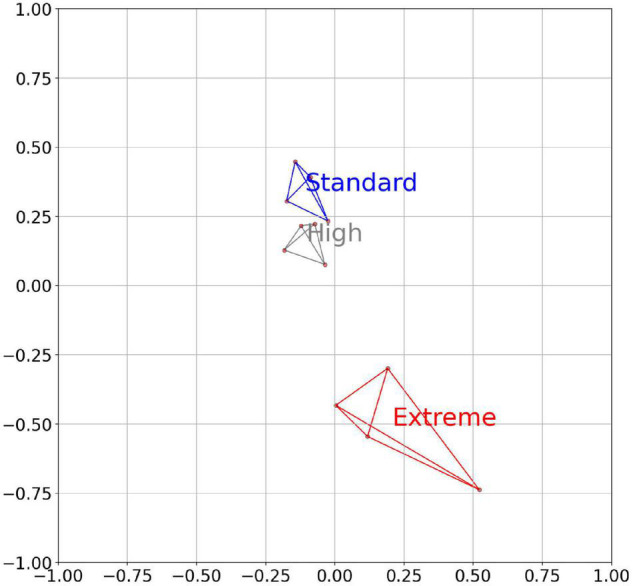
An MDS plot of the individual cows based on the relative abundance of the linkage targets across all significantly changed bins of the rumen metagenome. The separation score between the Standard and the Extreme is 2.11. The axes are arbitrary values, resulting from the reduction of the multidimensional space of correlation similarities into two dimensions.

In a similar way to the clustering based on bins (Figure 1) or the CAZy families (Figure 2), the cows could be separated into their respective feeding groups based on the predicted linkage target of the CAZymes encoded in the metagenome (Figure 3). Again, the abundance of linkage targets in the Standard and High feed groups were clustering closer together than the Extreme group.

**FIGURE 4:**
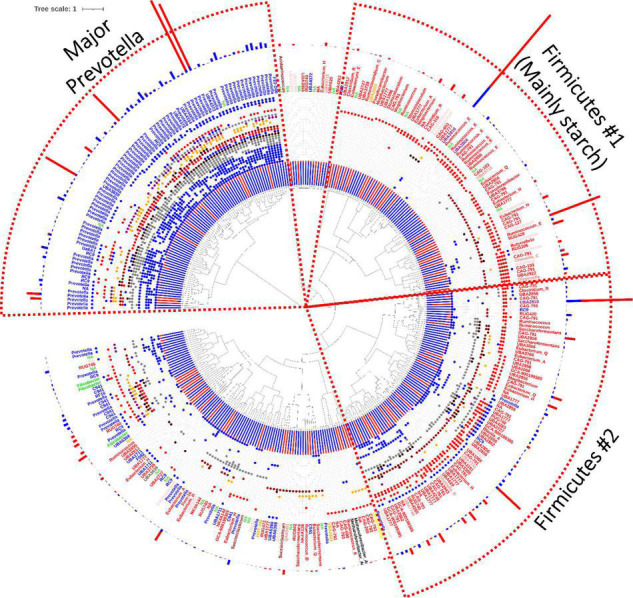
A dendrogram of the 348 bins found in the microbiome, which had a significant change in abundance between the Standard and the Extreme feeding groups (*t*-test, *p*-value < 0.05). In the dendrogram (in the center of the Figure), the bins are clustered based on the presence or absence of enzymes targeting different polysaccharide linkages, a novel approach to analyzing enzyme profile relatedness. The inner red and black-stacked bar plot indicates the relative abundance of the individual bin in the Standard (red) and the Extreme (black) groups. The 26 dots in different colors are indicating if a specific linkage target is present (colored) or absent (white). Pectin (blue), xylan (gray), starch (red), inulin (purple), other glucans (orange), cellulose (dark red), xyloglucan (brown). Only those 26 linkages found to be significantly changed between the Standard and the Extreme groups are included (the linkage targets included in Table 5 and continued in Table 6). The linkage targets are in the same order as those in Tables 5, 6, starting with “Pectin::Arabinogalactan::α-1,3/α-1,5::Ara” closest to the center. The small font text label states the assigned genus of the bin, whereas the color is according to the phylum of the bin, including Actinobacteriota (coral), Archaeal phyla (black), Bacteroidota (blue), Chloroflexota (turkish), Cyanobacteria (pink), Desulfobacterota_A (yellow), Fibrobacterota (green), Firmicutes (red), Patescibacteria (orange), Proteobacteria (purple), Verrucomicrobiota (brown). The outermost stacked bar plot indicates the relative abundance between bins (the very large bars indicate the more abundant bins in the metagenome for the Standard (blue) and the Extreme (red) groups. The tree can be interacted with on the link: https://itol.embl.de/tree/192381597419081638222742#.

To display which bins contributed with which linkage targets, a dendrogram was created in which the similarity (a Jaccard similarity coefficient score), was computed through a binary observation matrix, comparing the presence of enzymes predicted to have a specific linkage target. In the dendrogram (Figure 4), the Major *Prevotella* cluster, constituted about 25% of all significantly changed bins in the microbiome. However, within this cluster, the majority of the individual bins were low in abundance and primarily present in the Standard feed group (almost a hundred). Additionally, only a few very highly abundant bins were found in this *Prevotella* cluster, which were primarily found in the Extreme feed group. All the bins in the Major *Prevotella* cluster were rich in CAZymes active on many different linkage targets in the feed. Even though the majority of the *Prevotella* bins were found in the Major *Prevotella* cluster, a few *Prevotella* bins (species) were observed outside of the major *Prevotella* cluster, as a result of their generally poorer CAZymes content. The other cluster named Firmicutes #1 (Mainly starch) in the right upper part of the dendrogram was dominated by Firmicutes that primarily encoded for starch-degrading enzymes. The bottom half of the dendrogram included a range of bins, but the cluster to the right (Firmicutes #2) was primarily rich in *Firmicutes*, encoding a range of different starch degrading and non-starch degrading CAZymes with relatively more in the Standard feed group. The dendrogram thus disclosed that the feed-induced alterations in the Holstein cow microbiome could be systematically described by the changes in the metagenome-encoded CAZymes active on polysaccharides present in the feed. Notably, the inclusion of a CAZymes analysis revealed that the feed-induced alterations in the microbiome, indeed, concerned both composition and function and, consequently, that the methane-lowering effect of the extreme feed (Table 2) was mirrored as a clear shift in the abundance and diversity of the rumen metagenome-encoded CAZymes.

Lastly, the “antithetical effect” was also evident on a genus level as visualized in the bin-CAZyme profile-generated dendrogram (Figure 4). An example of this was obvious for the significantly altered abundance levels of the two species belonging to genus *RUG420* (Firmicutes): One was found almost exclusively in the Standard diet group, whereas the other was found almost exclusively in the Extreme diet group. Interestingly, these two species were in two different clusters, primarily containing Firmicutes (Figure 4); the one dominant in the Standard feed group was thus located in the Firmicutes #1 mainly starch cluster (the upper right cluster), whereas the other species, which was relatively more dominant in the Extreme feed group, was found in the Firmicutes #2 cluster in the dendrogram (Figure 4).

## Discussion

Comparison of the abundance of the bins within each of the feeding groups revealed major changes when summarized on a phyla level (Table 3). Surprisingly, the abundance change of the two Archaea phyla was not significant between the feeding groups. This observation is, in fact, in complete agreement with other recently reported data on rumen microbial community changes for Holstein cows-fed diets of varying concentrate-to-forage ratios (Noel et al., 2019). Evidently, the Archaea do not encode any relevant CAZymes, corroborating that they are not primary degraders of the feed fibers and feed carbohydrates in the rumen. Overall, the data also showed that feed-induced changes led to the majority of the bacterial phyla being most abundant in the Extreme feed, including Actinobacteriota, Desulfobacterota_A, the three Firmicutes, and Proteobacteria. However, a deeper analysis of the changes of the individual bins within each of the phyla clearly showed that the majority of the individual bins were more abundant in the Standard feed group, despite the overall trends. This “antithetical” phenomenon was due to the microbial diversity being larger in the Standard feed, whereas the microbiome in the Extreme feed group was largely dominated by fewer, more abundant single species (bins; Table 3). The fewer, more abundant species concur with the higher level of starch, i.e., inherently a higher abundance of enzymes attacking α-1,4-linked glucan in the extreme feed at the expense of more complex plant cell wall polysaccharides contained in the more fibrous, high-forage standard diet; the more complex polysaccharides in the standard diet led to a higher abundance of CAZymes, attacking β-1,4 xylan linkages, signifying the xylan backbone (Table 5). This change in the microbial composition was shown to be congruent with a shift in the CAZymes profile – going from a diverse “fiber-degrading” carbohydrate-processing enzyme profile toward a profile richer in starch-degrading CAZymes, specifically the following observations corroborated the higher complexity of the fibers in the standard feed: Higher abundance in the standard feed group of microbiome CAZymes, targeting (i) α-1,5 arabinan linkages, characteristic for arabinogalactan side chains of pectin, (ii) various canonical linkage types of both heteroxylan and pectin, including (iii) specific backbone substitutions, such as 4-methyl-glucuronolyation (α-1,2), feruloylation (ester), acetylation (ester), and methylation (Tables 5, 6).

A deeper look at the functional profile of the CAZymes, resolved in relation to the carbohydrate-linkage targets (Tables 5, 6), indeed, disclosed that the abundance of CAZymes-targeting linkages in complex hemicellulose structures, notably substituted β-1,4-linked heteroxylan (glucuronoarabinoxylan), was abundant in the rumen microbiomes of cows fed the more forage-rich feed (the Standard diet). This finding agrees with very recent data from Canada (Badhan et al., 2022); in that study, cow fecal material was analyzed by gene expression profiling and CAZyme family annotation, and high levels of CAZymes predicted to cleave the primary linkages within heteroxylan (as well as arabinan) were found. However, the findings in the present study (Table 5) that the Extreme feed was accompanied by a significant decrease in the CAZymes, targeting particular linkages of complex glucuronoarabinoxylan, are novel.

When considering the data in relation to the monosaccharide composition (Table 1), it is important to notice that the more extreme feed had a relatively high arabinose content compared to the xylose content, i.e., the arabinose increased despite the decrease in xylose. However, the xylose decrease was accompanied by an increase in the uronic acid content, indicative of pectin (Table 1). Although the abundancy of CAZymes attacking α-1,3/α-1,5 decreased with extreme feeding, the relative abundance increased CAZymes targeting arabinose attached to what we suggest being arabinogalactan (Table 6). The α-1,3 arabinose linkages may relate to either pectin or arabinoxylan, and these observations, together, help explain that the arabinose:xylose ratio in Table 1 increased despite the overall arabinoxylan level in the extreme feed being lower than in the standard feed. Furthermore, the relatively low level of arabinose in the standard feed is a direct consequence of the low arabinose:xylose ratio reported in corn (corn silage) and grass/clover silage compared to that in wheat (Rønn et al., 2022a).

We found that the diversity of carbohydrate-active enzymes active on fibers was lower in the high concentrate diet groups, which is in agreement with other recent data (Zhao et al., 2020). They found that carbohydrate esterases, in general, decrease when the ratio of concentrate increases, but they did not report which specific families or molecular functions these may have. In the present work, alterations within esterases, including acetyl-, feruloyl-, and methyl-esterases, appeared to contribute to the observed CAZyme alterations. Less-fiber-active esterase activity in the Extreme diet group may infer that less acetic acid, ferulic acid, and, particularly, methanol are released from the feed to the rumen fluid. Such a change would likely influence the activity of methylotrophic methanogens, causing them to form less methane. Changes within the carbohydrate-active enzymes were observed previously (Wang et al., 2019) but not with such a nuanced functional annotation to the substrate and not linked to significant changes. It has been proposed that substitutions on xylans are depressing fermentation of xylan fibers (Titgemeyer et al., 1991). Furthermore, it has been highlighted that acetyl xylanesterases are expressed in a great quantity for degradation of feed fibers (Badhan et al., 2022), which is found in many feed ingredients, including grasses (Biely et al., 2016). In summary, the data obtained thus suggest that the feed type is programming the rumen microbiome composition *via* selection of the microbiome’s enzymatic feed degradation capability. Indeed, peptide-based functional annotation by CUPP of the metagenome predicted CAZymes made directly on the total assembled metagenome database of the rumen microbiome samples provided a nuanced picture, and confirmed that feed-induced methane lowering was driven by a change in the microbiome correlated with the carbohydrate-processing capability of the organisms *via* their encoded CAZymes (Barrett and Lange, 2019). In addition to the direct enzymatic carbohydrate degradation, the volatiles also changed in response to the altered feed and altered microbial metabolism. Hence, as indicated in the Introduction, the acetate to propionate ratio was decreased from 2.70 in the rumen of the Standard feed group to 2.15 in the High feed group and to 1.55 in the Extreme feed group (Olijhoek et al., 2022).

The dendrogram clustering the individual bins based on the encoded carbohydrate-active enzymes and their predicted molecular functions did, indeed, organize the bins into their respective phyla, in general, for the three highlighted clusters. We have previously observed that it was possible to taxonomically organize certain fungi based on their predicted fiber-active carbohydrate-active enzymes (Barrett et al., 2020b), but, for bacteria, the separation is less obvious for the analyzed bacterial bins.

The members of Bacteroidota generally have a more diverse arsenal of fiber-active enzymes, whereas the Firmicutes seemed to form two distinct clusters. The first Firmicutes cluster (Figure 4 – Firmicutes #1) consisted primarily of starch active enzymes, whereas the second (Figure 4 – Firmicutes #2) had both starch-active enzymes and some general fiber-active enzymes. Investigation of the specific genome-encoded CAZymes in, for example, two RUG420 species, and their predicted polysaccharide enzyme linkage targets, revealed that both had the same number of pectin-active enzymes (2) and no xylan-active enymes. However, the species found in the Extreme feed group had more CAZymes predicted to be active on cellulose and starch (33% more), belonging to family GH3 and GH13, respectively. This difference in the CAZymes thus separated the two species into two different clusters. Uniquely, in the case of *Solobacterium sp900314345*, which was dominant in the Extreme feed group, a taxonomic counterpart also existed in the Standard group, but with unknown species id (bin 587). When inspecting the CAZymes, the species which was most dominant in the Standard group had seven enzymes predicted to be active on pectin, whereas the Extreme feed counterpart had only two. As discussed above, the reduction in pectin-active enzymes in the Extreme feed group included lower capability to remove methylation from pectin (*via* pectin methyl esterase; Table 5), directly reducing methanol formation, thus presumably limiting the metabolism of methylotrophic methanogens. Furthermore, three xylan-active enzymes were predicted to be encoded by the species dominant in the Standard group, whereas the species dominant in the Extreme group had none. The three xylan-active enzymes were predicted to be specific as two GH43 enzymes and one CE2 esterase were annotated. Moreover, the *UBA2856* spp. dominant in the Standard feed group, similarly, had a counterpart in the same genus (*UBA2856 sp900319065*). When the CAZymes were compared, the species dominant in the Standard feed group encoded more enzymes predicted to be active on pectin, xylan, and cellulose, whereas the enzymes expected to be active on starch were consistently high (12 CAZymes predicted to be active on starch). The completeness of the eight bins was 82 to 97.2%.

## Conclusion

The abundance of the individual species (bins) of the rumen fluid microbiome differed significantly between the Standard and the Extreme diets, whereas differences between the Standard and High were less profound. The bacterial species/bins mostly abundant in the Standard feed group nearly vanished in the Extreme group when analyzed at the phylum, genus or bin level, but the Archaea did generally not change, even though two bins of the 14 archaeal bins were significantly more abundant in the Standard diet group. Comparison of the predicted CAZymes abundancy based on the rumen metagenome DNA sequence analysis revealed a very different CAZyme arsenal in the Standard and the Extreme rumen microbiomes. Notably, the CAZymes predicted to be active on starch were relatively more abundant in the bins dominant in the Extreme diet group, whereas CAZymes predicted to be active on prevailing grass fibers, such as β-1,4-linked xylan and substituted glucuronoarabinoxylan, were significantly more abundant in the high methane producing the Standard diet group. Using a Spearman correlation measure, the abundance of these enzymes in the microbiome was positively correlated with the measured methane emission. By considering that the CH_4_ emission decreased with the more extreme feeding, we, therefore, interpret that the abundance of xylan-modifying enzymes (Table 5) in the microbiome decreased with the CH_4_ emission. The analytical approach provides a new way of performing rumen microbiome functional analysis. Overall, there was good compliance between the microbiome and methane data. The results of the CAZyme profile characterization of rumen microbiomes, sampled from Holstein cows fed standard vs. extreme diets, thus clearly confirmed a correlation between the rumen metagenome-encoded CAZymes and feed-induced changes in the cows’ enteric CH4 emission.

## Data Availability Statement

The datasets presented in this study can be found in online repositories. The name of the repository and accession number can be found below: EMBL-EBI; ERZ4932357.

## Author Contributions

CB carried out the *in vivo* experiment, including measurements of emissions, feed composition, and feed intake, and provided the rumen samples from the cattle for further processing. CB, DO, and PL were involved in conceptualized of the animal trial, the subsequent data analysis, and interpretation of results. KB processed the assembled bin data and annotated CAZy families. AM and LL supervised the work and the data interpretations of the metagenome annotations. KB and AM wrote the manuscript. All authors contributed to the article and approved the submitted version.

## Conflict of Interest

LL is employed by LLa-Bioeconomy. The remaining authors declare that the research was conducted in the absence of any commercial or financial relationships that could be construed as a potential conflict of interest.

## Publisher’s Note

All claims expressed in this article are solely those of the authors and do not necessarily represent those of their affiliated organizations, or those of the publisher, the editors and the reviewers. Any product that may be evaluated in this article, or claim that may be made by its manufacturer, is not guaranteed or endorsed by the publisher.
