# Supplementary material for: Changes in the Metagenome-Encoded CAZymes of the Rumen Microbiome Are Linked to Feed-Induced Reductions in Methane Emission From Holstein Cows
Source: Front Microbiol. 2022 May 20;13:855590. doi: 10.3389/fmicb.2022.855590 (PMC9163818; doi:10.3389/fmicb.2022.855590)
Supplement: Supplementary file 1 [file Data_Sheet_1.docx]

Supplementary material

Changes in the metagenome-encoded CAZymes of the rumen microbiome are linked to feed induced reductions in methane emission from Holstein cows

Kristian Barrett^1^, Lene Lange^2^, Christian F. Børsting^3^, Dana W. Olijhoek^3^, Peter Lund^3^, and Anne S. Meyer^1*^

^1^Protein Chemistry and Enzyme Technology Section, DTU Bioengineering, Department of Biotechnology and Biomedicine, Technical University of Denmark, 2800 Kgs. Lyngby, Denmark

^2^LLa-Bioeconomy, Research & Advisory, 2500 Valby, Denmark

^3^Department of Animal Science, AU Foulum, Aarhus University, 8830 Tjele, Denmark

*** Correspondence:** Corresponding Author, asme@dtu.dk

Supplementary Table 1 – The proportions of the different ingredients and the chemical composition of diets fed to lactating Holstein cows (g/kg of dry matter, DM) (Olijhoek et al., 2021).

|  | Diet | | |
| --- | --- | --- | --- |
| Item | Standard | High | Extreme |
| Ingredient composition |  |  |  |
| First and second cut grass/clover silage | 255 | 128 | - |
| Corn silage | 243 | 121 | - |
| Barley straw | 12.6 | 50.2 | 87.9 |
| Commercial concentrate mixture | 109 | 109 | 109 |
| Beet pulp, dried | 120 | 160 | 201 |
| Barley | 112 | 121 | 130 |
| Wheat, NaOH treated | - | 77.4 | 155 |
| Distillers grain, dried | - | 68.7 | 138 |
| Rapeseed cake | 78.6 | 106 | 134 |
| Soybean meal | 53.8 | 27.1 | - |
| Molasses (sugarcane) | 4.14 | 12.5 | 20.9 |
| Palm fatty acids distillate | 2.11 | 2.87 | 3.64 |
| Others (vitamins, minerals, salt, titanium dioxide) | 10.3 | 16.0 | 21.7 |
| Chemical composition |  |  |  |
| Ash | 58.1 | 60.8 | 63.1 |
| Crude protein | 159 | 164 | 171 |
| Crude fat | 36 | 39 | 42 |
| Starch | 173 | 194 | 223 |
| Neutral Detergent Fiber | 306 | 278 | 248 |
| Indigestible Neutral Detergent Fiber | 76.4 | 78.0 | 78.2 |

Supplementary Table 2 – The linkage targets of the predicted CAZy family and molecular function.

| **CAZy** | **EC** | **Substrate Class** | **Attack site** | **Bond broken** | **Released compound** |
| --- | --- | --- | --- | --- | --- |
| AA3 | **GENERAL** | Cellulose |  |  |  |
| AA3 | **1.1.99.18** | Cellulose |  |  |  |
| AA3 | **1.1.3.4** | Cellulose |  |  |  |
| AA3 | **1.1.3.7** | Cellulose |  |  |  |
| AA3 | **1.1.3.13** | Cellulose |  |  |  |
| AA3 | **1.1.3.10** | Cellulose |  |  |  |
| AA9 | **GENERAL** | Cellulose | [Glc]n::[Glc]n | C1/C4 | [Glc]n |
| AA9 | **1.14.99.54** | Cellulose | [Glc]n::[Glc]n | C1 | [Glc]n |
| AA9 | **1.14.99.56** | Cellulose | [Glc]n::[Glc]n | C4 | [Glc]n |
| AA10 | **GENERAL** | Cellulose | [Glc]n::[Glc]n | C1/C4 | [Glc]n |
| AA10 | **1.14.99.54** | Cellulose | [Glc]n::[Glc]n | C1 | [Glc]n |
| AA10 | **1.14.99.56** | Cellulose | [Glc]n::[Glc]n | C4 | [Glc]n |
| AA13 | **GENERAL** | Starch |  |  |  |
| AA13 | **1.14.99.55** | Starch |  |  |  |
| AA14 | **GENERAL** | Xylan | [Xyl]n::[Xyl]n | ? | [Xyl]n |
| AA14 | **1.*.*.*** | Xylan | [Xyl]n::[Xyl]n | ? | [Xyl]n |
| AA15 | **GENERAL** | Cellulose | [Glc]n::[Glc]n | C1 | [Glc]n |
| AA15 | **1.14.99.54** | Cellulose | [Glc]n::[Glc]n | C1 | [Glc]n |
| AA16 | **GENERAL** | Cellulose | [Glc]n::[Glc]n | C1 | [Glc]n |
| AA16 | **1.14.99.54** | Cellulose | [Glc]n::[Glc]n | C1 | [Glc]n |
| CE0 | **3.1.1.11** | Pectin-HG |  |  | Acetyl/Methyl |
| CE1 | **3.1.1.72** | Xylan |  |  | Methyl |
| CE1 | **3.1.1.73** | Xylan |  |  | Ferulate |
| CE2 | **GENERAL** | Xylan |  |  | Methyl |
| CE2 | **3.1.1.72** | Xylan |  |  | Methyl |
| CE3 | **GENERAL** | Xylan |  |  | Acetyl |
| CE3 | **3.1.1.72** | Xylan |  |  | Acetyl |
| CE4 | **3.1.1.72** | Xylan |  |  | Acetyl |
| CE5 | **3.1.1.72** | Xylan |  |  | Acetyl |
| CE6 | **GENERAL** | Xylan |  |  | Acetyl |
| CE6 | **3.1.1.72** | Xylan |  |  | Acetyl |
| CE7 | **GENERAL** | Xylan |  |  | Acetyl |
| CE7 | **3.1.1.72** | Xylan |  |  | Acetyl |
| CE8 | **GENERAL** | Pectin-HG |  |  | Methyl |
| CE8 | **3.1.1.11** | Pectin-HG |  |  | Methyl |
| CE12 | **3.1.1.*** | Pectin-HG |  |  | Acetyl |
| CE12 | **3.1.1.72** | Xylan |  |  | Acetyl |
| CE13 | **GENERAL** | Pectin-HG |  |  | Acetyl |
| CE13 | **3.1.1.*** | Pectin-HG |  |  | Acetyl |
| CE15 | **GENERAL** | Xylan |  |  | Methyl |
| CE15 | **3.1.1.*** | Xylan-GX |  |  | Methyl |
| CE16 | **GENERAL** | Xylan |  |  | Acetyl |
| CE16 | **3.1.1.6** | Xylan |  |  | Acetyl |
| CE17 | **GENERAL** | Mannan |  |  | Acetyl |
| CE17 | **3.1.1.*** | Mannan |  |  | Acetyl |
| GH0 | **3.2.1.23** | Pectin-AGI | Gal::[Gal]n:: | β-1,4 | Gal |
| GH0 | **3.2.1.4** | Cellulose | [Glc]n::[Glc]n | β-1,4 | [Glc]n |
| GH0 | **3.2.1.21** | Cellulose | [Glc]n::[Glc]n | β-1,4 | Glc |
| GH1 | **3.2.1.21** | Cellulose | [Glc]n::[Glc]n | β-1,4 | Glc |
| GH1 | **3.2.1.23** | Pectin-AGI | Gal::[Gal]n:: | β-1,4 | Gal |
| GH1 | **3.2.1.25** | Mannan |  |  | Man |
| GH1 | **3.2.1.37** | Xylan | Xyl::[Xyl]n::[Xyl]n | β-1,4 | Xyl |
| GH1 | **3.2.1.38** | Seaweed |  |  | (D)Fuc |
| GH1 | **3.2.1.*** | Cellulose | [Glc]n::[Glc]n | β-1,4 | Glc |
| GH2 | **3.2.1.23** | Pectin-AGI | Gal::[Gal]n:: | β-1,4 | Gal |
| GH2 | **3.2.1.25** | Mannan |  |  | Man |
| GH2 | **3.2.1.31** | Pectin-RGII | ::Fuc::GlcA | β-1,4 | GlcA |
| GH2 | **3.2.1.55** | Pectin-AG | Ara::[Ara]n:: | α-1,3/α-1,5 | Ara |
| GH2 | **3.2.1.*** | Pectin-RGII | ::Gal::Ara(p) | α-1,4 | Ara(p) |
| GH2 | **3.2.1.*** | Pectin-RGII | ::Fuc::GalA | β-1,3 | GalA |
| GH2 | **3.2.1.37** | Xylan | Xyl::[Xyl]n::[Xyl]n | β-1,4 | Xyl |
| GH3 | **3.2.1.21** | Cellulose | Glc::[Glc]n | β-1,4 | Glc |
| GH3 | **3.2.1.37** | Xylan | Xyl::[Xyl]n::[Xyl]n | β-1,4 | Xyl |
| GH3 | **3.2.1.55** | Pectin-AG | Ara::[Ara]n:: | α-1,3/α-1,5 | Ara |
| GH3 | **3.2.1.58** | Glucan | [Glc]n::[Glc]n | β-1,3 | Glc |
| GH3 | **3.2.1.74** | Cellulose | [Glc]n::[Glc]n | β-1,4 | Glc |
| GH3 | **3.2.1.120** | Xyloglucan |  |  |  |
| GH4 | **3.2.1.20** | Starch |  |  |  |
| GH5 | **3.2.1.4** | Cellulose | [Glc]n::[Glc]n | β-1,4 | [Glc]n |
| GH5 | **3.2.1.8** | Xylan | [Xyl]n::[Xyl]n | β-1,4 | [Xyl]n |
| GH5 | **3.2.1.21** | Cellulose | Glc::[Glc]n | β-1,4 | Glc |
| GH5 | **3.2.1.25** | Mannan |  |  | Man |
| GH5 | **3.2.1.58** | Glucan | [Glc]n::[Glc]n | β-1,3 | Glc |
| GH5 | **3.2.1.74** | Cellulose | [Glc]n::Glc | β-1,4 |  |
| GH5 | **3.2.1.75** | Glucan | [Glc]n::[Glc]n | β-1,6 | Glc |
| GH5 | **3.2.1.78** | Mannan | [Man]n::[Man]n | β-1,4 | Man |
| GH5 | **3.2.1.91** | Cellulose | Glc::Glc::[Glc]n | β-1,4 | Glc::Glc |
| GH5 | **3.2.1.151** | Xyloglucan |  |  |  |
| GH5 | **3.2.1.164** | Pectin-AGII |  | β-1,6 | Gal |
| GH5 | **2.4.1.*** | Mannan |  |  |  |
| GH5 | **3.2.1.73** | Glucan | [Glc]n::[Glc]n | β-1,3(4) | [Glc]n |
| GH5 | **3.2.1.39** | Glucan | [Glc]n::[Glc]n | β-1,3 | [Glc]n |
| GH6 | **GENERAL** | Cellulose | [Glc]n::[Glc]n | β-1,4 |  |
| GH6 | **3.2.1.4** | Cellulose | [Glc]n::[Glc]n | β-1,4 | [Glc]n |
| GH6 | **3.2.1.91** | Cellulose | Glc::Glc::[Glc]n | β-1,4 | Glc::Glc |
| GH6 | **3.2.1.73** | Glucan | [Glc]n::[Glc]n | β-1,3(4) | [Glc]n |
| GH7 | **3.2.1.4** | Cellulose | [Glc]n::[Glc]n | β-1,4 | [Glc]n |
| GH7 | **3.2.1.176** | Cellulose | [Glc]n::Glc::Glc | β-1,4 | Glc::Glc |
| GH7 | **3.2.1.73** | Glucan | [Glc]n::[Glc]n | β-1,3(4) | [Glc]n |
| GH8 | **3.2.1.4** | Cellulose | [Glc]n::[Glc]n | β-1,4 | [Glc]n |
| GH8 | **3.2.1.73** | Glucan | [Glc]n::[Glc]n | β-1,3/β-1,4 | [Glc]n |
| GH8 | **3.2.1.8** | Xylan | [Xyl]n::[Xyl]n | β-1,4 | [Xyl]n |
| GH8 | **3.2.1.156** | Xylan | [Xyl]n::Xyl | β-1,4 | Xyl |
| GH9 | **3.2.1.4** | Cellulose | [Glc]n::[Glc]n | β-1,4 | [Glc]n |
| GH9 | **3.2.1.6** | Glucan | [Glc]n::[Glc]n | β-1,3(4) | [Glc]n |
| GH9 | **3.2.1.21** | Cellulose | [Glc]n::[Glc]n | β-1,4 | Glc |
| GH9 | **3.2.1.73** | Glucan | [Glc]n::[Glc]n | β-1,3(4) | Glc |
| GH9 | **3.2.1.74** | Cellulose | [Glc]n::[Glc]n | β-1,4 | Glc |
| GH9 | **3.2.1.91** | Cellulose | Glc::Glc::[Glc]n | β-1,4 | Glc::Glc |
| GH9 | **3.2.1.151** | Xyloglucan |  |  | Glc |
| GH10 | **3.2.1.8** | Xylan | [Xyl]n::[Xyl]n | β-1,4 | [Xyl]n |
| GH10 | **3.2.1.32** | Xylan | [Xyl]n::[Xyl]n | β-1,3 | [Xyl]n |
| GH10 | **2.4.2.*** | Xylan-Trans |  |  |  |
| GH10 | **3.2.1.4** | Cellulose | [Glc]n::[Glc]n | β-1,4 | [Glc]n |
| GH11 | **GENERAL** | Xylan |  | β-1,4 | Xyl |
| GH11 | **3.2.1.8** | Xylan | [Xyl]n::[Xyl]n | β-1,4 | [Xyl]n |
| GH11 | **3.2.1.32** | Xylan | [Xyl]n::[Xyl]n | β-1,3 | [Xyl]n |
| GH12 | **3.2.1.4** | Cellulose | [Glc]n::[Glc]n | β-1,4 | [Glc]n |
| GH12 | **3.2.1.151** | Xyloglucan |  |  | Glc |
| GH12 | **3.2.1.73** | Glucan | [Glc]n::[Glc]n | β-1,3/β-1,4 | [Glc]n |
| GH12 | **2.4.1.207** | Xyloglucan |  |  | Glc |
| GH13 | **GENERAL** | Starch | [Glc]n::[Glc]n |  | Glc |
| GH13 | **3.2.1.1** | Starch | [Glc]n::[Glc]n | α-1,4 | [Glc]n::[Glc]n |
| GH13 | **3.2.1.41** | Starch | [Glc]n::[Glc]n | α-1,6 | Glc::Glc |
| GH13 | **2.4.1.19** | Starch |  |  | Glc |
| GH13 | **3.2.1.54** | Starch | [Glc]n::[Glc]n | α-1,6 | Glc |
| GH13 | **3.2.1.93** | Starch |  |  | Phosphate |
| GH13 | **3.2.1.10** | Starch | [Glc]n::[Glc]n | α-1,6 | Glc::Glc |
| GH13 | **3.2.1.133** | Starch | Glc::Glc::[Glc]n::[Glc]n | α-1,4 | Glc::Glc |
| GH13 | **3.2.1.135** | Starch | [Glc]n::[Glc]n |  | [Glc]n::[Glc]n |
| GH13 | **3.2.1.20** | Starch | Glc::[Glc]n::[Glc]n | α-1,4 | Glc |
| GH13 | **3.2.1.60** | Starch | Glc::Glc::Glc::[Glc]n::[Glc]n | α-1,4 | Glc::Glc::Glc |
| GH13 | **3.2.1.68** | Starch | [Glc]n::[Glc]n | α-1,6 | Glc::Glc |
| GH13 | **3.2.1.70** | Starch | [Glc]n::[Glc]n | α-1,6 | Glc |
| GH13 | **3.2.1.98** | Starch | Glc::Glc::[Glc]n::[Glc]n | α-1,4 | Glc::Glc |
| GH13 | **3.2.1.116** | Starch | Glc::Glc::[Glc]n::[Glc]n | α-1,4 | Glc::Glc |
| GH13 | **2.4.1.18** | Starch |  |  | Glc+ |
| GH13 | **5.4.99.16** | Starch |  |  | Glc+ |
| GH13 | **2.4.1.25** | Starch |  |  | Glc+ |
| GH13 | **3.2.1.*** | Starch | Glc::Glc::[Glc]n::[Glc]n | α-1,4 | Glc::Glc |
| GH13 | **2.4.1.4** | Starch | Glc::Glc | α-1,4 | Glc |
| GH13 | **2.4.1.7** | Starch | Glc::Glc[P} |  | Phosphate |
| GH13 | **3.2.1.141** | Starch | Glc::Glc::Glc::[Glc]n::[Glc]n | α-1,4 | Glc::Glc::Glc |
| GH13 | **5.4.99.11** | Starch |  |  | Glc+ |
| GH13 | **5.4.99.15** | Starch |  |  | Glc+ |
| GH13 | **3.2.1.33** | Starch | [Glc]n::[Glc]n(Glc) | α-1,6 | Glc |
| GH13 | **2.4.99.16** | Starch |  |  | Phosphate |
| GH13 | **2.4.1.329** | Starch |  |  | Phosphate |
| GH13 | **2.4.1.359** | Starch |  |  | Phosphate |
| GH14 | **GENERAL** | Starch |  |  |  |
| GH14 | **3.2.1.2** | Starch | Glc::Glc::[Glc]n::[Glc]n | α-1,4 | Glc::Glc |
| GH15 | **GENERAL** | Starch | [Glc]n::[Glc]n |  | Glc |
| GH15 | **3.2.1.3** | Starch | Glc::[Glc]n::[Glc]n | α-1,4 | Glc |
| GH15 | **3.2.1.70** | Starch | [Glc]n::[Glc]n | α-1,6 | Glc |
| GH15 | **3.2.1.28** | Glucan | Glc::Glc | α-1,1 | Glc |
| GH15 | **2.4.1.2** | Starch | Glc::Glc::[Glc]n::[Glc]n | α-1,4 | Glc::Glc |
| GH16 | **2.4.1.207** | Xyloglucan |  |  |  |
| GH16 | **3.2.1.39** | Glucan | [Glc]n::[Glc]n | β-1,3 | [Glc]n |
| GH16 | **3.2.1.6** | Glucan | [Glc]n::[Glc]n | β-1,3/β-1,4 | [Glc]n |
| GH16 | **3.2.1.73** | Glucan | [Glc]n::[Glc]n | β-1,3(4) | [Glc]n |
| GH16 | **3.2.1.81** | Seaweed |  |  |  |
| GH16 | **3.2.1.83** | Seaweed |  |  |  |
| GH16 | **3.2.1.151** | Xyloglucan |  |  |  |
| GH16 | **3.2.1.181** | Pectin-AGII |  | β-1,3 | Gal |
| GH16 | **3.2.1.*** | Seaweed |  |  |  |
| GH17 | **GENERAL** | Glucan |  |  |  |
| GH17 | **3.2.1.39** | Glucan | [Glc]n::[Glc]n | β-1,3 | [Glc]n |
| GH17 | **3.2.1.58** | Glucan | Glc::[Glc]n::[Glc]n | β-1,3 | Glc |
| GH17 | **3.2.1.73** | Glucan | [Glc]n::[Glc]n | β-1,3(4) | [Glc]n |
| GH17 | **3.2.1.175** | Glucan |  |  |  |
| GH17 | **2.4.1.*** | Glucan |  | β-1,3 |  |
| GH26 | **3.2.1.78** | Mannan |  |  | Man |
| GH26 | **3.2.1.100** | Mannan |  |  | Man |
| GH26 | **3.2.1.32** | Xylan | [Xyl]n::[Xyl]n | β-1,3 | [Xyl]n |
| GH26 | **3.2.1.*** | Mannan |  |  |  |
| GH27 | **3.2.1.22** | Pectin-AGP | ::Ara(f)::Gal | α-1,3 | Gal |
| GH28 | **GENERAL** | Pectin |  |  |  |
| GH28 | **3.2.1.15** | Pectin-HG | [GalA]n::[GalA]n | ? | [GalA]n |
| GH28 | **3.2.1.40** | Pectin-RGII | ? | α- | Rha |
| GH28 | **3.2.1.67** | Pectin-HG | GalA::[GalA]n | α-1,4 | GalA |
| GH28 | **3.2.1.82** | Pectin-HG | GalA::GalA::[GalA]n | α-1,4 | GalA::GalA |
| GH28 | **3.2.1.171** | Pectin-RGII | ? | ? | ? |
| GH28 | **3.2.1.173** | Pectin-RGI |  |  |  |
| GH28 | **3.2.1.*** | Pectin-XG |  | α-1,4 | GalA{Xyl} |
| GH29 | **3.2.1.51** | Xyloglucan | ::Fuc | α- | Fuc |
| GH30 | **3.2.1.8** | Xylan | [Xyl]n::[Xyl]n | β-1,4 | [Xyl]n |
| GH30 | **3.2.1.21** | Cellulose | [Glc]n::[Glc]n | β-1,4 | Glc |
| GH30 | **3.2.1.37** | Xylan | Xyl::[Xyl]n::[Xyl]n | β-1,4 | Xyl |
| GH30 | **3.2.1.75** | Glucan | [Glc]n::[Glc]n | β-1,6 | [Glc]n |
| GH30 | **3.2.1.136** | Xylan-AX | ? | β-1,4 | ? |
| GH30 | **3.2.1.164** | Pectin-AGII |  | β-1,6 | Gal |
| GH30 | **3.2.1.*** | Xylan | [Xyl]n::Xyl | ? | Xyl |
| GH31 | **3.2.1.20** | Starch | Glc::[Glc]n::[Glc]n | α-1,4 | Glc |
| GH31 | **3.2.1.24** | Mannan |  |  |  |
| GH31 | **3.2.1.84** | Glucan | [Glc]n::[Glc]n | α-1,3 | Glc |
| GH31 | **3.2.1.10** | Glucan | [Glc]n::[Glc]n | α-1,6 | Glc |
| GH31 | **3.2.1.177** | Xyloglucan | Xyl::[Glc]n | α- | Xyl |
| GH31 | **4.2.2.13** | Starch | Glc::[Glc]n::[Glc]n | α-1,4 | Glc |
| GH31 | **3.2.1.48** | Starch | Glc::Fru | α-1,2 | Glc |
| GH33 | **3.2.1.*** | Pectin-RGII |  |  | Kdo |
| GH35 | **3.2.1.23** | Pectin-AGI | Gal::[Gal]n:: | β-1,4 | Gal |
| GH35 | **3.2.1.*** | Pectin-AG |  | β-1,3/β-1,4 | Gal |
| GH37 | **GENERAL** | Glucan |  |  |  |
| GH37 | **3.2.1.28** | Glucan | Glc::Glc | α-1,1 | Glc |
| GH39 | **3.2.1.37** | Xylan | Xyl::[Xyl]n::[Xyl]n | β-1,4 | Xyl |
| GH42 | **3.2.1.23** | Pectin-AGI | Gal::[Gal]n:: | β-1,4 | Gal |
| GH42 | **3.2.1.*** | Pectin-RGII | Gal::Ara(p) | α-1,4 | Ara(p) |
| GH43 | **3.2.1.37** | Xylan | Xyl::[Xyl]n::[Xyl]n | β-1,4 | Xyl |
| GH43 | **3.2.1.55** | Pectin-AG | Ara::[Ara]n:: | α-1,3/α-1,5 | Ara |
| GH43 | **3.2.1.8** | Xylan | [Xyl]n::[Xyl]n | β-1,4 | [Xyl]n |
| GH43 | **3.2.1.*** | Xylan | [Xyl]n::[Xyl]n | β-1,3 | Xyl |
| GH43 | **3.2.1.*** | Pectin-AG |  |  | Ara |
| GH43 | **3.2.1.99** | Pectin-AGII |  | α-1,5 | Ara |
| GH43 | **3.2.1.145** | Pectin-AGII |  | β-1,3 | Gal |
| GH44 | **3.2.1.4** | Cellulose | [Glc]n::[Glc]n | β-1,4 | [Glc]n |
| GH44 | **3.2.1.151** | Xyloglucan |  |  |  |
| GH45 | **3.2.1.4** | Cellulose | [Glc]n::[Glc]n | β-1,4 | [Glc]n |
| GH45 | **3.2.1.151** | Xyloglucan | [Glc]n::{Xyl}Glc::[Glc]n | ? | {Xyl}Glc::[Glc]n |
| GH45 | **3.2.1.78** | Mannan | [Man]n::[Man]n | β-1,4 | Man |
| GH48 | **3.2.1.176** | Cellulose | [Glc]n::Glc::Glc | β-1,4 | Glc::Glc |
| GH48 | **3.2.1.4** | Cellulose | [Glc]n::[Glc]n | β-1,4 | ? |
| GH49 | **3.2.1.57** | Glucan |  |  |  |
| GH50 | **GENERAL** | Seaweed-Agar |  |  |  |
| GH50 | **3.2.1.81** | Seaweed-Agar |  |  |  |
| GH51 | **3.2.1.4** | Cellulose | [Glc]n::[Glc]n | β-1,4 | [Glc]n |
| GH51 | **3.2.1.8** | Xylan | [Xyl]n::[Xyl]n | β-1,4 | [Xyl]n |
| GH51 | **3.2.1.37** | Xylan | Xyl::[Xyl]n::[Xyl]n | β-1,4 | Xyl |
| GH51 | **3.2.1.55** | Pectin-AG | Ara::[Ara]n:: | α-1,3/α-1,5 | Ara |
| GH51 | **3.2.1.73** | Glucan | [Glc]n::[Glc]n | β-1,3(4) | [Glc]n |
| GH52 | **GENERAL** | Xylan |  | β-1,4 | Xyl |
| GH52 | **3.2.1.37** | Xylan | Xyl::[Xyl]n::[Xyl]n | β-1,4 | Xyl |
| GH53 | **GENERAL** | Pectin-AGI | ? | β-1,4 | Gal |
| GH53 | **3.2.1.89** | Pectin-AGI | ? | β-1,4 | Gal |
| GH54 | **GENERAL** | Xylan-AX |  |  |  |
| GH54 | **3.2.1.55** | Pectin-AG | Ara::[Ara]n:: | α-1,3/α-1,5 | Ara |
| GH54 | **3.2.1.37** | Xylan | Xyl::[Xyl]n::[Xyl]n | β-1,4 | Xyl |
| GH55 | **GENERAL** | Glucan | [Glc]n::[Glc]n | β-1,3 | Glc |
| GH55 | **3.2.1.58** | Glucan | [Glc]n::[Glc]n | β-1,3 | Glc |
| GH55 | **3.2.1.39** | Glucan | [Glc]n::[Glc]n | β-1,3 | [Glc]n |
| GH57 | **3.2.1.1** | Starch | [Glc]n::[Glc]n | α-1,4 | [Glc]n::[Glc]n |
| GH57 | **3.2.1.41** | Glucan | [Glc]n::[Glc]n | α-1,6 | Glc::Glc |
| GH57 | **3.2.1.54** | Glucan | [Glc]n::[Glc]n:: | α-1,4 | [Glc]n::[Glc]n |
| GH57 | **2.4.1.18** | Glucan |  |  |  |
| GH57 | **2.4.1.25** | Glucan |  |  |  |
| GH59 | **3.2.1.23** | Pectin-AGI | Gal::[Gal]n:: | β-1,4 | Gal |
| GH62 | **GENERAL** | Xylan-AX |  | α-1,3/α-1,5 | Ara |
| GH62 | **3.2.1.55** | Pectin-AG | Ara::[Ara]n:: | α-1,3/α-1,5 | Ara |
| GH63 | **3.2.1.84** | Glucan | [Glc]n::[Glc]n | α-1,3 | Glc |
| GH63 | **3.2.1.20** | Starch | Glc::[Glc]n::[Glc]n | α-1,4 | Glc |
| GH64 | **GENERAL** | Glucan | [Glc]n::[Glc]n | β-1,3 | Glc |
| GH64 | **3.2.1.39** | Glucan | [Glc]n::[Glc]n | β-1,3 | Glc |
| GH65 | **3.2.1.28** | Glucan |  |  |  |
| GH67 | **GENERAL** | Xylan-GX |  |  |  |
| GH67 | **3.2.1.139** | Xylan-GX | [Xyl]n::[Xyl]n(GlcA) |  | GlcA |
| GH67 | **3.2.1.131** | Xylan-GX | [Xyl]n::[Xyl]n(GlcA) | α-1,2 | GlcA |
| GH71 | **GENERAL** | Glucan |  |  |  |
| GH71 | **3.2.1.59** | Glucan |  | α-1,3 |  |
| GH74 | **GENERAL** | Xyloglucan |  |  |  |
| GH74 | **3.2.1.4** | Cellulose | [Glc]n::[Glc]n | β-1,4 | [Glc]n |
| GH74 | **3.2.1.150** | Xyloglucan |  |  |  |
| GH74 | **3.2.1.151** | Xyloglucan |  |  |  |
| GH76 | **3.2.1.20** | Starch | Glc::[Glc]n::[Glc]n | α-1,4 | Glc |
| GH78 | **GENERAL** | Pectin |  |  |  |
| GH78 | **3.2.1.40** | Pectin-RGII |  | α- | Rha |
| GH78 | **3.2.1.174** | Pectin-RGI | Rha::[GalA::Rha]n | α-1,4 | Rha |
| GH78 | **3.2.1.*** | Pectin-RGII | ::Api::Rha |  | Api+Rha |
| GH81 | **GENERAL** | Glucan |  |  |  |
| GH81 | **3.2.1.39** | Glucan | [Glc]n::[Glc]n | β-1,3 | [Glc]n |
| GH82 | **GENERAL** | Seaweed-Carrageenan |  |  |  |
| GH82 | **3.2.1.157** | Seaweed-Carrageenan |  |  |  |
| GH86 | **3.2.1.81** | Seaweed-Agar |  |  |  |
| GH87 | **GENERAL** | Glucan |  |  |  |
| GH87 | **3.2.1.61** | Glucan | [Glc]n::[Glc]n | α-1,3(4) | [Glc]n |
| GH87 | **3.2.1.59** | Glucan | [Glc]n::[Glc]n | α-1,3 | [Glc]n |
| GH88 | **GENERAL** | Seaweed-Ulvan |  |  |  |
| GH88 | **3.2.1.*** | Seaweed-Ulvan |  |  |  |
| GH90 | **GENERAL** | Seaweed-Rhamnan |  |  |  |
| GH90 | **3.2.1.*** | Seaweed-Rhamnan |  |  |  |
| GH93 | **GENERAL** | Pectin-AGII | ? | α-1,5 | Ara |
| GH93 | **3.2.1.*** | Pectin-AGII | ? | α-1,5 | Ara |
| GH95 | **3.2.1.51** | Xyloglucan | ::Fuc | α- | Fuc |
| GH95 | **3.2.1.63** | Xyloglucan | ::Fuc | α-1,2 | Fuc |
| GH95 | **3.2.1.*** | Pectin-RGII | ::Fuc::GlcA::(L) | α-1,2 | (L)Gal |
| GH96 | **GENERAL** | Seaweed-Agar |  |  |  |
| GH96 | **3.2.1.158** | Seaweed-Agar |  |  |  |
| GH97 | **3.2.1.3** | Starch | Glc::[Glc]n::[Glc]n | α-1,4 | Glc |
| GH97 | **3.2.1.20** | Starch | Glc::[Glc]n::[Glc]n | α-1,4 | Glc |
| GH98 | **3.2.1.8** | Xylan | [Xyl]n::[Xyl]n | β-1,4 | [Xyl]n |
| GH105 | **3.2.1.172** | Pectin-HG | GalA::GalA | α-1,4 | Rha |
| GH105 | **3.2.1.*** | Pectin-HG | GalA::GalA | α-1,4 | Rha |
| GH105 | **3.2.1.*** | Seaweed-Ulvan |  |  |  |
| GH106 | **GENERAL** | Pectin-RGII |  | α- | Rha |
| GH106 | **3.2.1.40** | Pectin-RGII |  | α- | Rha |
| GH106 | **3.2.1.174** | Pectin-RGI | Rha::[GalA::Rha]n | α-1,4 | Rha |
| GH107 | **GENERAL** | Seaweed |  |  |  |
| GH107 | **3.2.1.*** | Seaweed |  |  |  |
| GH113 | **GENERAL** | Mannan |  |  |  |
| GH113 | **3.2.1.78** | Mannan | [Man]n::[Man]n | ? | Man |
| GH115 | **GENERAL** | Xylan-GX | [Xyl]n::[Xyl]n(GlcA) | α-1,2 | GlcA |
| GH115 | **3.2.1.131** | Xylan-GX | [Xyl]n::[Xyl]n(GlcA) | α-1,2 | GlcA |
| GH115 | **3.2.1.*** | Xylan-GX | [Xyl]n::[Xyl]n(GlcA) | α-1,2 | (4-O-methyl)::GlcA |
| GH116 | **3.2.1.21** | Cellulose | [Glc]n::[Glc]n | β-1,4 | Glc |
| GH116 | **3.2.1.37** | Xylan | Xyl::[Xyl]n::[Xyl]n | β-1,4 | Xyl |
| GH117 | **GENERAL** | Seaweed-Agar |  |  |  |
| GH117 | **3.2.1.*** | Seaweed-Agar |  |  |  |
| GH118 | **GENERAL** | Seaweed-Agar |  |  |  |
| GH118 | **3.2.1.81** | Seaweed-Agar |  |  |  |
| GH119 | **GENERAL** | Starch |  |  |  |
| GH119 | **3.2.1.1** | Starch | [Glc]n::[Glc]n | α-1,4 | [Glc]n::[Glc]n |
| GH120 | **3.2.1.37** | Xylan | Xyl::[Xyl]n::[Xyl]n | β-1,4 | Xyl |
| GH122 | **GENERAL** | Starch |  |  |  |
| GH122 | **3.2.1.20** | Starch | Glc::[Glc]n::[Glc]n | α-1,4 | Glc |
| GH124 | **GENERAL** | Cellulose | [Glc]n::[Glc]n | β-1,4 | [Glc]n |
| GH124 | **3.2.1.4** | Cellulose | [Glc]n::[Glc]n | β-1,4 | [Glc]n |
| GH126 | **GENERAL** | Starch | [Glc]n::[Glc]n | α-1,4 | Glc |
| GH126 | **3.2.1.*** | Starch | [Glc]n::[Glc]n | α-1,4 | Glc |
| GH127 | **3.2.1.185** | Pectin-AG |  | β-1,2 | Ara |
| GH127 | **3.2.1.*** | Pectin-RGII | ::Api::Rha::Ace | α-1,3 | Api::Rha |
| GH128 | **GENERAL** | Glucan | [Glc]n::[Glc]n | β-1,3 | [Glc]n |
| GH128 | **3.2.1.39** | Glucan | [Glc]n::[Glc]n | β-1,3 | [Glc]n |
| GH131 | **GENERAL** | Glucan |  |  |  |
| GH131 | **3.2.1.*** | Glucan | [Glc]n::[Glc]n | β-1,3/β-1,6 | [Glc]n |
| GH132 | **GENERAL** | Glucan |  | β-1,3 |  |
| GH132 | **3.2.1.*** | Glucan | [Glc]n::[Glc]n | β-1,3 | [Glc]n |
| GH133 | **GENERAL** | Glucan |  |  |  |
| GH134 | **GENERAL** | Mannan | [Man]n::[Man]n | β-1,4 | [Man]n |
| GH134 | **3.2.1.78** | Mannan | [Man]n::[Man]n | β-1,4 | [Man]n |
| GH137 | **3.2.1.185** | Pectin-RGII | ::Ara(p)::Rha::Ara(f) | β-1,2 | Ara |
| GH137 | **3.2.1.185** | Pectin-AG |  | β-1,2 | Ara |
| GH138 | **GENERAL** | Pectin-RGI | GalA::[Rha::GalA]n | α-1,2 | GalA |
| GH138 | **3.2.1.173** | Pectin-RGI | GalA::[Rha::GalA]n | α-1,2 | GalA |
| GH139 | **GENERAL** | Pectin-RGII | Api::Rha::Ace::Gal::{2Me}Fuc | α-1,2 | {2Me}Fuc |
| GH139 | **3.2.1.*** | Pectin-RGII | Api::Rha::Ace::Gal::{2Me}Fuc | α-1,2 | {2Me}Fuc |
| GH140 | **GENERAL** | Pectin-RGII | GalA::Api::Rha | β-1,2 | Api::Rha |
| GH140 | **3.2.1.*** | Pectin-RGII | GalA::Api::Rha | β-1,2 | Api::Rha |
| GH141 | **3.2.1.51** | Pectin-RGII | ::Rha::Fuc::{2Me}Xyl | α-1,4 | Fuc::{2Me}Xyl |
| GH141 | **3.2.1.8** | Xylan | [Xyl]n::[Xyl]n | β-1,4 | [Xyl]n |
| GH142 | **GENERAL** | Pectin-RGII | [GalA]n::Dha::Ara(f) | β-1,5 | Ara |
| GH142 | **3.2.1.185** | Pectin-RGII | [GalA]n::Dha::Ara(f) | β-1,5 | Ara |
| GH143 | **GENERAL** | Pectin-RGII | [GalA]n::Dha::Ara(f) | β-2,3 | Dha::Ara(f) |
| GH143 | **3.2.1.*** | Pectin-RGII | [GalA]n::Dha::Ara(f) | β-2,3 | Dha::Ara(f) |
| GH144 | **GENERAL** | Glucan | [Glc]n::[Glc]n | β-1,2 | [Glc]n |
| GH144 | **3.2.1.71** | Glucan | [Glc]n::[Glc]n | β-1,2 | [Glc]n |
| GH144 | **3.2.1.*** | Glucan | [Glc]n::[Glc]n | β-1,2 | [Glc]n |
| GH145 | **GENERAL** | Pectin-AGP | ::Gal::GlcA::Rha | α-1,4 | Rha |
| GH145 | **3.2.1.*** | Pectin-AGP | ::Gal::GlcA::Rha | α-1,4 | Rha |
| GH146 | **3.2.1.185** | Pectin-RGII | [GalA]n::Dha::Ara(f) | β-1,2 | Ara |
| GH146 | **3.2.1.185** | Pectin-RGII | ::Ara(p)::Rha::Ara(f) | β-1,2 | Ara |
| GH147 | **GENERAL** | Pectin-AG |  |  | Gal |
| GH147 | **3.2.1.23** | Pectin-AGI | Gal::[Gal]n:: | β-1,4 | Gal |
| GH148 | **GENERAL** | Glucan |  | β-1,3 |  |
| GH148 | **3.2.1.*** | Glucan | [Glc]n::[Glc]n | β-1,3 | [Glc]n |
| GH150 | **GENERAL** | Seaweed-Carrageenan |  |  |  |
| GH150 | **3.2.1.*** | Seaweed-Carrageenan |  |  |  |
| GH151 | **3.2.1.51** | Xyloglucan | ::Fuc | α- | Fuc |
| GH152 | **GENERAL** | Glucan |  |  |  |
| GH152 | **3.2.1.39** | Glucan | [Glc]n::[Glc]n | β-1,3 | [Glc]n |
| GH154 | **GENERAL** | Seaweed |  |  |  |
| GH154 | **3.2.1.31** | Pectin-AGP |  | β-1,6 | GlcA |
| GH157 | **GENERAL** | Glucan |  |  |  |
| GH157 | **3.2.1.39** | Glucan | [Glc]n::[Glc]n | β-1,3 | [Glc]n |
| GH158 | **GENERAL** | Glucan |  |  |  |
| GH158 | **3.2.1.39** | Glucan | [Glc]n::[Glc]n | β-1,3 | [Glc]n |
| GH160 | **GENERAL** | Pectin-AGI |  | β-1,4 | Gal |
| GH160 | **3.2.1.*** | Pectin-AGI |  | β-1,4 | Gal |
| GH162 | **GENERAL** | Glucan |  |  |  |
| GH162 | **3.2.1.71** | Glucan | [Glc]n::[Glc]n | β-1,2 | [Glc]n |
| GH164 | **GENERAL** | Mannan |  |  |  |
| GH164 | **3.2.1.25** | Mannan |  |  |  |
| GH165 | **GENERAL** | Pectin-AG |  |  |  |
| GH165 | **3.2.1.23** | Pectin-AGI | Gal::[Gal]n:: | β-1,4 | Gal |
| PL0 | **4.2.2.3** | Seaweed-Alginate |  |  |  |
| PL1 | **GENERAL** | Pectin-HG |  | α-1,4 | (D4)GalA |
| PL1 | **4.2.2.2** | Pectin-HG |  | α-1,4 | (D4)GalA |
| PL1 | **4.2.2.9** | Pectin-HG | [GalA]n::GalA::GalA | α-1,4 | (D4)GalA::GalA |
| PL1 | **4.2.2.10** | Pectin-HG | [GalA]n::(Me)GalA::[GalA]n | α-1,4 | (D4-Me)GalA::[GalA]n |
| PL2 | **GENERAL** | Pectin-HG |  | α-1,4 | (D4)GalA |
| PL2 | **4.2.2.2** | Pectin-HG | [GalA]n::[GalA]n | α-1,4 | (D4)GalA::[GalA]n |
| PL2 | **4.2.2.9** | Pectin-HG | [GalA]n::GalA::GalA | α-1,4 | (D4)GalA::GalA |
| PL3 | **GENERAL** | Pectin-HG |  | α-1,4 | (D4)GalA::[GalA]n |
| PL3 | **4.2.2.2** | Pectin-HG | [GalA]n::[GalA]n | α-1,4 | (D4)GalA::[GalA]n |
| PL4 | **GENERAL** | Pectin-RGI |  | α-1,4 | (D4)GalA::Rha::[GalA::Rha]n-1 |
| PL4 | **4.2.2.23** | Pectin-RGI | [GalA::Rha]n::[GalA::Rha]n | α-1,4 | (D4)GalA::Rha::[GalA::Rha]n-1 |
| PL5 | **GENERAL** | Seaweed-Alginate |  |  |  |
| PL5 | **4.2.2.3** | Seaweed-Alginate |  |  |  |
| PL5 | **4.2.2.14** | Seaweed-Alginate |  |  |  |
| PL6 | **GENERAL** | Seaweed-Alginate |  |  |  |
| PL6 | **4.2.2.3** | Seaweed-Alginate |  |  |  |
| PL6 | **4.2.2.*** | Seaweed-Alginate |  |  |  |
| PL6 | **4.2.2.11** | Seaweed-Alginate |  |  |  |
| PL6 | **4.2.2.26** | Seaweed-Alginate |  |  |  |
| PL7 | **GENERAL** | Seaweed-Alginate |  |  |  |
| PL7 | **4.2.2.3** | Seaweed-Alginate |  |  |  |
| PL7 | **4.2.2.11** | Seaweed-Alginate |  |  |  |
| PL7 | **4.2.2.*** | Seaweed-Alginate |  |  |  |
| PL7 | **4.2.2.14** | Seaweed-Alginate |  |  |  |
| PL7 | **4.2.2.26** | Seaweed-Alginate |  |  |  |
| PL9 | **GENERAL** | Pectin |  |  |  |
| PL9 | **4.2.2.2** | Pectin-HG | [GalA]n::[GalA]n | α-1,4 | (D4)GalA::[GalA]n |
| PL9 | **4.2.2.9** | Pectin-HG | [GalA]n::GalA::GalA | α-1,4 | (D4)GalA::GalA |
| PL9 | **4.2.2.23** | Pectin-RGI | [GalA::Rha]n::[GalA::Rha]n | α-1,4 | (D4)GalA::Rha::[GalA::Rha]n-1 |
| PL10 | **GENERAL** | Pectin-HG |  | α-1,4 | (D4)GalA::[GalA]n |
| PL10 | **4.2.2.2** | Pectin-HG | [GalA]n::[GalA]n | α-1,4 | (D4)GalA::[GalA]n |
| PL11 | **GENERAL** | Pectin-RGI |  | α-1,4 | GalA |
| PL11 | **4.2.2.23** | Pectin-RGI | [GalA::Rha]n::[GalA::Rha]n | α-1,4 | (D4)GalA::Rha::[GalA::Rha]n-1 |
| PL11 | **4.2.2.24** | Pectin-RGI | (D4)GalA::Rha::GalA::Rha | α-1,4 | (D4)GalA::Rha |
| PL14 | **GENERAL** | Seaweed-Alginate |  |  |  |
| PL14 | **4.2.2.3** | Seaweed-Alginate |  |  |  |
| PL14 | **4.2.2.26** | Seaweed-Alginate |  |  |  |
| PL14 | **4.2.2.14** | Seaweed-Alginate |  |  |  |
| PL14 | **4.2.2.*** | Seaweed-Alginate |  |  |  |
| PL15 | **4.2.2.3** | Seaweed-Alginate |  |  |  |
| PL15 | **4.2.2.26** | Seaweed-Alginate |  |  |  |
| PL17 | **GENERAL** | Seaweed-Alginate |  |  |  |
| PL17 | **4.2.2.3** | Seaweed-Alginate |  |  |  |
| PL17 | **4.2.2.26** | Seaweed-Alginate |  |  |  |
| PL18 | **GENERAL** | Seaweed-Alginate |  |  |  |
| PL18 | **4.2.2.3** | Seaweed-Alginate |  |  |  |
| PL18 | **4.2.2.11** | Seaweed-Alginate |  |  |  |
| PL18 | **4.2.2.*** | Seaweed-Alginate |  |  |  |
| PL20 | **GENERAL** | Seaweed-Alginate |  |  |  |
| PL20 | **4.2.2.14** | Seaweed-Alginate |  |  |  |
| PL22 | **GENERAL** | Pectin-HG | GalA::GalA | α-1,4 | GalA |
| PL22 | **4.2.2.6** | Pectin-HG | GalA::GalA | α-1,4 | GalA |
| PL24 | **GENERAL** | Seaweed-Ulvan |  |  |  |
| PL24 | **4.2.2.*** | Seaweed-Ulvan |  |  |  |
| PL25 | **GENERAL** | Seaweed-Ulvan |  |  |  |
| PL25 | **4.2.2.*** | Seaweed-Ulvan |  |  |  |
| PL26 | **GENERAL** | Pectin-RGI |  | α-1,4 | (D4)GalA::Rha |
| PL26 | **4.2.2.24** | Pectin-RGI | (D4)GalA::Rha::GalA::Rha | α-1,4 | (D4)GalA::Rha |
| PL27 | **GENERAL** | Pectin-AGP | ::GlcA::Rha | α-1,4 | Rha |
| PL27 | **4.2.2.*** | Pectin-AGP | ::GlcA::Rha | α-1,4 | Rha |
| PL28 | **GENERAL** | Seaweed-Ulvan |  |  |  |
| PL28 | **4.2.2.*** | Seaweed-Ulvan |  |  |  |
| PL31 | **GENERAL** | Seaweed-Alginate |  |  |  |
| PL31 | **4.2.2.14** | Seaweed-Alginate |  |  |  |
| PL31 | **4.2.2.3** | Seaweed-Alginate |  |  |  |
| PL32 | **GENERAL** | Seaweed-Alginate |  |  |  |
| PL32 | **4.2.2.3** | Seaweed-Alginate |  |  |  |
| PL34 | **GENERAL** | Seaweed-Alginate |  |  |  |
| PL34 | **4.2.2.*** | Seaweed-Alginate |  |  |  |
| PL36 | **GENERAL** | Seaweed-Alginate |  |  |  |
| PL36 | **4.2.2.3** | Seaweed-Alginate |  |  |  |
| PL37 | **4.2.2.*** | Seaweed-Ulvan |  |  |  |
| PL38 | **GENERAL** | Seaweed-Alginate |  |  |  |
| PL38 | **4.2.2.14** | Seaweed-Alginate |  |  |  |
| PL39 | **GENERAL** | Seaweed-Alginate |  |  |  |
| PL39 | **4.2.2.*** | Seaweed-Alginate |  |  |  |
| PL40 | **GENERAL** | Seaweed-Ulvan |  |  |  |
| PL40 | **4.2.2.*** | Seaweed-Ulvan |  |  |  |

| Supplementary Table 3 – The individual bins and their individual abundance change between the diet groups. | | | | | | | | | | | |
| --- | --- | --- | --- | --- | --- | --- | --- | --- | --- | --- | --- |
| **bin** | **Comp.  (%)** | **Cont. (%)** | **Genus** | **Species** | **Average Standard** | **Average Extreme** | **p-value** | **Log2 Fold Change** | **Standard** | **Extreme** | **No change** |
| 564 | 29.68 | 4.46 | g__ | s__ | 0.036 | 0.016 | 0.967 | 1.146 |  |  | 1 |
| 47 | 89.09 | 0 | g__Methanobrevibacter | s__ | 0.037 | 0.018 | 0.834 | 1.015 |  |  | 1 |
| 460 | 98.13 | 5.07 | g__Methanobrevibacter | s__Methanobrevibacter sp900314635 | 0.466 | 0.202 | 0.960 | 1.208 |  |  | 1 |
| 37 | 79.91 | 24.45 | g__Methanobrevibacter_A | s__ | 0.088 | 0.003 | 0.028 | 4.955 | 1 |  |  |
| 143 | 22.43 | 3.74 | g__Methanobrevibacter_A | s__ | 0.005 | 0.005 | 0.192 | -0.046 |  |  | 1 |
| 659 | 89.72 | 31.97 | g__Methanobrevibacter_A | s__ | 0.046 | 0.089 | 0.097 | -0.965 |  |  | 1 |
| 660 | 41.59 | 4.67 | g__Methanobrevibacter_A | s__ | 0.010 | 0.010 | 0.512 | -0.053 |  |  | 1 |
| 661 | 100.00 | 16.88 | g__Methanobrevibacter_A | s__ | 0.059 | 0.039 | 0.471 | 0.613 |  |  | 1 |
| 705 | 18.79 | 0.86 | g__Methanobrevibacter_A | s__ | 0.050 | 0.001 | 0.015 | 5.204 | 1 |  |  |
| 43 | 59.83 | 6.8 | g__Methanobrevibacter_A | s__Methanobrevibacter_A sp900314615 | 0.027 | 0.004 | 0.079 | 2.595 |  |  | 1 |
| 930 | 79.44 | 28.34 | g__Methanobrevibacter_A | s__Methanobrevibacter_A sp900314695 | 0.050 | 0.006 | 0.132 | 3.103 |  |  | 1 |
| 704 | 29.44 | 1.87 | g__Methanobrevibacter_A | s__Methanobrevibacter_A sp900320515 | 0.018 | 0.001 | 0.091 | 5.135 |  |  | 1 |
| 697 | 82.36 | 31.87 | g__Methanosphaera | s__ | 0.042 | 0.054 | 0.291 | -0.349 |  |  | 1 |
| 67 | 94.39 | 21.5 | g__Methanosphaera | s__Methanosphaera sp11921u | 0.043 | 0.071 | 0.106 | -0.729 |  |  | 1 |
| 925 | 34.69 | 0 | g__ | s__ | 0.021 | 0.004 | 0.093 | 2.488 |  |  | 1 |
| 643 | 95.62 | 11.32 | g__Bifidobacterium | s__Bifidobacterium merycicum | 0.661 | 0.167 | 0.566 | 1.985 |  |  | 1 |
| 402 | 29.31 | 0 | g__Bifidobacterium | s__Bifidobacterium ruminantium | 0.107 | 0.041 | 0.888 | 1.377 |  |  | 1 |
| 658 | 27.59 | 0 | g__Bifidobacterium | s__Bifidobacterium ruminantium | 0.021 | 0.009 | 0.928 | 1.312 |  |  | 1 |
| 325 | 100.00 | 0 | g__Olegusella | s__Olegusella sp900314685 | 0.096 | 7.851 | 0.063 | -6.360 |  |  | 1 |
| 651 | 63.15 | 1.21 | g__Olegusella | s__Olegusella sp900315165 | 0.004 | 0.183 | 0.000 | -5.501 |  | 1 |  |
| 749 | 97.58 | 12.1 | g__Olsenella_B | s__ | 0.009 | 0.795 | 0.017 | -6.526 |  | 1 |  |
| 553 | 39.09 | 0.92 | g__Olsenella_C | s__ | 0.020 | 0.005 | 0.508 | 2.099 |  |  | 1 |
| 202 | 88.97 | 19.77 | g__Olsenella_C | s__Olsenella_C umbonata | 0.181 | 0.263 | 0.017 | -0.538 |  | 1 |  |
| 200 | 77.15 | 0 | g__Pseudoscardovia | s__Pseudoscardovia suis | 0.052 | 0.083 | 0.369 | -0.677 |  |  | 1 |
| 176 | 99.78 | 0.81 | g__QAMH01 | s__ | 0.008 | 0.354 | 0.007 | -5.404 |  | 1 |  |
| 254 | 86.81 | 3.75 | g__RUG033 | s__RUG033 sp900314665 | 0.004 | 0.128 | 0.000 | -4.854 |  | 1 |  |
| 668 | 84.11 | 0.2 | g__RUG844 | s__RUG844 sp900313875 | 0.119 | 0.057 | 0.790 | 1.056 |  |  | 1 |
| 397 | 53.92 | 30.17 | g__UBA1367 | s__ | 0.084 | 0.014 | 0.021 | 2.607 | 1 |  |  |
| 498 | 96.37 | 1.88 | g__UBA7741 | s__UBA7741 sp900314495 | 0.425 | 2.337 | 0.047 | -2.459 |  | 1 |  |
| 638 | 94.35 | 0.81 | g__UBA7748 | s__UBA7748 sp900314535 | 0.008 | 0.467 | 0.011 | -5.782 |  | 1 |  |
| 404 | 41.38 | 6.43 | g__UBA9715 | s__ | 0.054 | 0.002 | 0.000 | 4.921 | 1 |  |  |
| 266 | 18.42 | 0 | g__UBA9715 | s__ | 0.011 | 0.000 | 0.000 | 5.059 | 1 |  |  |
| 590 | 73.84 | 3.67 | g__ | s__ | 0.433 | 0.002 | 0.003 | 7.959 | 1 |  |  |
| 284 | 78.19 | 3.84 | g__C941 | s__ | 0.266 | 0.004 | 0.242 | 6.237 |  |  | 1 |
| 757 | 50.00 | 25 | g__C941 | s__ | 0.140 | 0.001 | 0.000 | 7.415 | 1 |  |  |
| 811 | 18.10 | 0 | g__C941 | s__ | 0.059 | 0.000 | 0.009 | 7.134 | 1 |  |  |
| 667 | 29.31 | 3.45 | g__C941 | s__ | 0.036 | 0.000 | 0.000 | 7.001 | 1 |  |  |
| 424 | 31.03 | 0 | g__C941 | s__ | 0.004 | 0.089 | 0.004 | -4.407 |  | 1 |  |
| 528 | 23.28 | 1.72 | g__C941 | s__C941 sp002395965 | 0.036 | 0.001 | 0.073 | 5.940 |  |  | 1 |
| 578 | 32.76 | 5.17 | g__C941 | s__C941 sp900318065 | 0.066 | 0.001 | 0.001 | 6.244 | 1 |  |  |
| 809 | 79.58 | 27.2 | g__C941 | s__C941 sp900318535 | 0.608 | 0.002 | 0.013 | 8.571 | 1 |  |  |
| 359 | 88.79 | 64.64 | g__C941 | s__C941 sp900320055 | 0.822 | 0.003 | 0.008 | 8.266 | 1 |  |  |
| 117 | 34.48 | 0 | g__C941 | s__C941 sp900321725 | 0.015 | 0.265 | 0.003 | -4.144 |  | 1 |  |
| 198 | 69.89 | 10.22 | g__F082 | s__ | 0.098 | 0.003 | 0.003 | 5.084 | 1 |  |  |
| 142 | 30.25 | 0 | g__F082 | s__ | 0.022 | 0.000 | 0.010 | 6.155 | 1 |  |  |
| 652 | 27.59 | 0 | g__F23-D06 | s__ | 0.014 | 0.003 | 0.053 | 2.400 |  |  | 1 |
| 802 | 18.97 | 0 | g__F23-D06 | s__F23-D06 sp900315245 | 0.015 | 0.000 | 0.000 | 5.690 | 1 |  |  |
| 601 | 62.16 | 1.04 | g__Ga6A1 | s__Ga6A1 sp900102385 | 0.254 | 0.001 | 0.004 | 8.075 | 1 |  |  |
| 433 | 76.86 | 44.28 | g__Prevotella | s__ | 1.669 | 0.060 | 0.070 | 4.791 |  |  | 1 |
| 468 | 83.86 | 95.77 | g__Prevotella | s__ | 1.252 | 0.013 | 0.000 | 6.638 | 1 |  |  |
| 306 | 31.74 | 12.89 | g__Prevotella | s__ | 1.184 | 0.044 | 0.000 | 4.736 | 1 |  |  |
| 417 | 63.78 | 46.55 | g__Prevotella | s__ | 1.138 | 0.034 | 0.000 | 5.055 | 1 |  |  |
| 356 | 97.18 | 143.81 | g__Prevotella | s__ | 1.095 | 0.015 | 0.000 | 6.182 | 1 |  |  |
| 544 | 71.75 | 40.29 | g__Prevotella | s__ | 1.090 | 0.082 | 0.065 | 3.734 |  |  | 1 |
| 370 | 69.75 | 36.44 | g__Prevotella | s__ | 1.055 | 0.022 | 0.000 | 5.594 | 1 |  |  |
| 557 | 90.38 | 49.36 | g__Prevotella | s__ | 1.038 | 0.020 | 0.023 | 5.705 | 1 |  |  |
| 896 | 85.83 | 7.8 | g__Prevotella | s__ | 0.987 | 1.565 | 0.087 | -0.665 |  |  | 1 |
| 822 | 45.10 | 7.5 | g__Prevotella | s__ | 0.917 | 0.037 | 0.089 | 4.640 |  |  | 1 |
| 220 | 83.36 | 26.08 | g__Prevotella | s__ | 0.797 | 0.003 | 0.007 | 7.837 | 1 |  |  |
| 126 | 75.12 | 13.4 | g__Prevotella | s__ | 0.696 | 0.013 | 0.000 | 5.714 | 1 |  |  |
| 229 | 86.54 | 69.51 | g__Prevotella | s__ | 0.675 | 0.003 | 0.000 | 7.616 | 1 |  |  |
| 502 | 47.02 | 10.34 | g__Prevotella | s__ | 0.603 | 0.011 | 0.027 | 5.815 | 1 |  |  |
| 455 | 41.38 | 8.62 | g__Prevotella | s__ | 0.576 | 0.009 | 0.000 | 5.999 | 1 |  |  |
| 277 | 78.30 | 24.2 | g__Prevotella | s__ | 0.560 | 0.048 | 0.050 | 3.530 |  |  | 1 |
| 565 | 37.93 | 10.34 | g__Prevotella | s__ | 0.545 | 0.007 | 0.000 | 6.382 | 1 |  |  |
| 211 | 35.27 | 16.67 | g__Prevotella | s__ | 0.526 | 0.782 | 0.009 | -0.571 |  | 1 |  |
| 399 | 65.50 | 29.95 | g__Prevotella | s__ | 0.474 | 0.018 | 0.000 | 4.731 | 1 |  |  |
| 566 | 79.85 | 13.7 | g__Prevotella | s__ | 0.432 | 0.050 | 0.076 | 3.117 |  |  | 1 |
| 572 | 54.31 | 5.79 | g__Prevotella | s__ | 0.410 | 0.015 | 0.001 | 4.811 | 1 |  |  |
| 294 | 40.28 | 10.34 | g__Prevotella | s__ | 0.391 | 0.008 | 0.000 | 5.576 | 1 |  |  |
| 260 | 95.75 | 4.18 | g__Prevotella | s__ | 0.365 | 0.014 | 0.007 | 4.752 | 1 |  |  |
| 256 | 58.62 | 8.62 | g__Prevotella | s__ | 0.342 | 0.002 | 0.004 | 7.197 | 1 |  |  |
| 60 | 79.56 | 21.62 | g__Prevotella | s__ | 0.332 | 0.079 | 0.194 | 2.076 |  |  | 1 |
| 320 | 88.60 | 8.6 | g__Prevotella | s__ | 0.308 | 0.255 | 0.448 | 0.275 |  |  | 1 |
| 302 | 50.50 | 10.77 | g__Prevotella | s__ | 0.308 | 0.002 | 0.001 | 7.124 | 1 |  |  |
| 512 | 55.25 | 23.2 | g__Prevotella | s__ | 0.292 | 0.006 | 0.001 | 5.632 | 1 |  |  |
| 400 | 43.26 | 6.9 | g__Prevotella | s__ | 0.265 | 0.030 | 0.007 | 3.122 | 1 |  |  |
| 22 | 21.16 | 1.72 | g__Prevotella | s__ | 0.246 | 0.002 | 0.000 | 6.955 | 1 |  |  |
| 392 | 12.23 | 3.45 | g__Prevotella | s__ | 0.246 | 0.653 | 0.078 | -1.410 |  |  | 1 |
| 206 | 45.28 | 4.94 | g__Prevotella | s__ | 0.244 | 0.022 | 0.001 | 3.471 | 1 |  |  |
| 868 | 82.76 | 16.51 | g__Prevotella | s__ | 0.239 | 0.001 | 0.000 | 7.679 | 1 |  |  |
| 167 | 49.14 | 13.79 | g__Prevotella | s__ | 0.235 | 0.002 | 0.000 | 6.707 | 1 |  |  |
| 332 | 60.18 | 34.63 | g__Prevotella | s__ | 0.229 | 0.008 | 0.000 | 4.905 | 1 |  |  |
| 353 | 61.81 | 15.52 | g__Prevotella | s__ | 0.225 | 0.006 | 0.000 | 5.183 | 1 |  |  |
| 807 | 55.33 | 0 | g__Prevotella | s__ | 0.207 | 0.003 | 0.096 | 6.307 |  |  | 1 |
| 333 | 66.36 | 2.35 | g__Prevotella | s__ | 0.204 | 0.001 | 0.004 | 7.135 | 1 |  |  |
| 831 | 47.76 | 16.85 | g__Prevotella | s__ | 0.197 | 0.076 | 0.762 | 1.376 |  |  | 1 |
| 141 | 74.62 | 9.73 | g__Prevotella | s__ | 0.197 | 0.002 | 0.005 | 6.405 | 1 |  |  |
| 28 | 43.10 | 5.17 | g__Prevotella | s__ | 0.192 | 0.006 | 0.081 | 5.038 |  |  | 1 |
| 311 | 42.34 | 2.04 | g__Prevotella | s__ | 0.186 | 0.002 | 0.001 | 6.939 | 1 |  |  |
| 734 | 20.69 | 0 | g__Prevotella | s__ | 0.179 | 0.001 | 0.000 | 7.738 | 1 |  |  |
| 649 | 13.17 | 1.72 | g__Prevotella | s__ | 0.177 | 0.015 | 0.107 | 3.607 |  |  | 1 |
| 300 | 57.21 | 6.32 | g__Prevotella | s__ | 0.162 | 0.002 | 0.003 | 6.283 | 1 |  |  |
| 768 | 34.14 | 10.34 | g__Prevotella | s__ | 0.153 | 0.008 | 0.010 | 4.246 | 1 |  |  |
| 613 | 63.79 | 0 | g__Prevotella | s__ | 0.149 | 0.002 | 0.001 | 6.314 | 1 |  |  |
| 689 | 47.70 | 6.9 | g__Prevotella | s__ | 0.139 | 0.006 | 0.000 | 4.606 | 1 |  |  |
| 372 | 10.34 | 0 | g__Prevotella | s__ | 0.132 | 0.221 | 0.064 | -0.749 |  |  | 1 |
| 177 | 20.69 | 0 | g__Prevotella | s__ | 0.129 | 0.002 | 0.024 | 6.168 | 1 |  |  |
| 97 | 27.59 | 6.9 | g__Prevotella | s__ | 0.119 | 0.004 | 0.029 | 4.954 | 1 |  |  |
| 297 | 20.69 | 5.17 | g__Prevotella | s__ | 0.096 | 0.000 | 0.042 | 7.595 | 1 |  |  |
| 430 | 38.95 | 6.92 | g__Prevotella | s__ | 0.091 | 0.003 | 0.000 | 5.079 | 1 |  |  |
| 84 | 20.61 | 0 | g__Prevotella | s__ | 0.089 | 0.002 | 0.000 | 5.691 | 1 |  |  |
| 203 | 13.79 | 1.72 | g__Prevotella | s__ | 0.078 | 0.001 | 0.046 | 6.501 | 1 |  |  |
| 817 | 56.39 | 1.48 | g__Prevotella | s__ | 0.077 | 0.078 | 0.355 | -0.015 |  |  | 1 |
| 86 | 10.34 | 1.72 | g__Prevotella | s__ | 0.075 | 0.000 | 0.000 | 8.021 | 1 |  |  |
| 770 | 34.20 | 0 | g__Prevotella | s__ | 0.067 | 0.001 | 0.000 | 6.171 | 1 |  |  |
| 184 | 15.52 | 0 | g__Prevotella | s__ | 0.060 | 0.000 | 0.003 | 7.927 | 1 |  |  |
| 769 | 29.31 | 0 | g__Prevotella | s__ | 0.059 | 0.001 | 0.001 | 5.378 | 1 |  |  |
| 929 | 17.24 | 0 | g__Prevotella | s__ | 0.057 | 0.005 | 0.003 | 3.412 | 1 |  |  |
| 427 | 36.21 | 0 | g__Prevotella | s__ | 0.056 | 0.001 | 0.000 | 5.405 | 1 |  |  |
| 23 | 19.44 | 0 | g__Prevotella | s__ | 0.056 | 0.001 | 0.000 | 5.976 | 1 |  |  |
| 670 | 22.41 | 0 | g__Prevotella | s__ | 0.050 | 0.000 | 0.005 | 7.261 | 1 |  |  |
| 786 | 16.51 | 0.19 | g__Prevotella | s__ | 0.035 | 0.000 | 0.002 | 7.525 | 1 |  |  |
| 687 | 24.14 | 0 | g__Prevotella | s__ | 0.034 | 0.016 | 0.919 | 1.124 |  |  | 1 |
| 182 | 12.38 | 0 | g__Prevotella | s__ | 0.033 | 0.015 | 0.905 | 1.110 |  |  | 1 |
| 830 | 51.72 | 0 | g__Prevotella | s__ | 0.031 | 0.015 | 0.783 | 1.059 |  |  | 1 |
| 313 | 13.79 | 1.72 | g__Prevotella | s__ | 0.030 | 0.000 | 0.002 | 7.825 | 1 |  |  |
| 327 | 71.28 | 3.65 | g__Prevotella | s__ | 0.026 | 0.317 | 0.017 | -3.626 |  | 1 |  |
| 759 | 18.97 | 1.72 | g__Prevotella | s__ | 0.023 | 0.001 | 0.000 | 5.285 | 1 |  |  |
| 380 | 15.52 | 0 | g__Prevotella | s__ | 0.020 | 0.107 | 0.040 | -2.405 |  | 1 |  |
| 410 | 10.34 | 0 | g__Prevotella | s__ | 0.017 | 0.002 | 0.038 | 2.952 | 1 |  |  |
| 692 | 12.07 | 0 | g__Prevotella | s__ | 0.011 | 0.011 | 0.342 | -0.034 |  |  | 1 |
| 495 | 25.86 | 0 | g__Prevotella | s__ | 0.009 | 0.038 | 0.006 | -1.998 |  | 1 |  |
| 422 | 36.21 | 0 | g__Prevotella | s__Prevotella bryantii | 0.392 | 0.032 | 0.000 | 3.612 | 1 |  |  |
| 362 | 48.19 | 1.21 | g__Prevotella | s__Prevotella bryantii | 0.187 | 0.014 | 0.000 | 3.750 | 1 |  |  |
| 683 | 70.69 | 0 | g__Prevotella | s__Prevotella copri | 0.005 | 0.090 | 0.176 | -4.113 |  |  | 1 |
| 920 | 92.40 | 9.46 | g__Prevotella | s__Prevotella sp000434515 | 0.014 | 0.180 | 0.001 | -3.689 |  | 1 |  |
| 50 | 84.55 | 42.1 | g__Prevotella | s__Prevotella sp002342665 | 1.442 | 0.041 | 0.006 | 5.153 | 1 |  |  |
| 912 | 90.92 | 54.32 | g__Prevotella | s__Prevotella sp002350355 | 0.813 | 5.315 | 0.012 | -2.709 |  | 1 |  |
| 425 | 90.54 | 55.56 | g__Prevotella | s__Prevotella sp002351725 | 1.565 | 0.013 | 0.000 | 6.942 | 1 |  |  |
| 928 | 83.75 | 14.67 | g__Prevotella | s__Prevotella sp002353585 | 0.678 | 0.003 | 0.037 | 7.614 | 1 |  |  |
| 336 | 54.67 | 12.9 | g__Prevotella | s__Prevotella sp002354095 | 0.333 | 0.008 | 0.005 | 5.373 | 1 |  |  |
| 836 | 85.65 | 4.31 | g__Prevotella | s__Prevotella sp002391185 | 0.347 | 0.016 | 0.004 | 4.418 | 1 |  |  |
| 405 | 83.59 | 3.74 | g__Prevotella | s__Prevotella sp002480935 | 0.029 | 0.688 | 0.034 | -4.547 |  | 1 |  |
| 585 | 77.74 | 17.4 | g__Prevotella | s__Prevotella sp900100635 | 0.500 | 0.083 | 0.024 | 2.587 | 1 |  |  |
| 195 | 60.80 | 8.3 | g__Prevotella | s__Prevotella sp900110085 | 0.274 | 0.002 | 0.000 | 6.850 | 1 |  |  |
| 420 | 67.99 | 1.46 | g__Prevotella | s__Prevotella sp900314455 | 0.022 | 0.848 | 0.010 | -5.287 |  | 1 |  |
| 910 | 13.95 | 0 | g__Prevotella | s__Prevotella sp900314715 | 0.063 | 0.001 | 0.028 | 6.763 | 1 |  |  |
| 265 | 63.79 | 20.69 | g__Prevotella | s__Prevotella sp900314755 | 0.450 | 0.051 | 0.007 | 3.134 | 1 |  |  |
| 255 | 83.14 | 1.42 | g__Prevotella | s__Prevotella sp900314915 | 0.030 | 0.038 | 0.258 | -0.354 |  |  | 1 |
| 252 | 70.69 | 34.48 | g__Prevotella | s__Prevotella sp900314935 | 0.621 | 0.071 | 0.002 | 3.132 | 1 |  |  |
| 439 | 83.22 | 4.35 | g__Prevotella | s__Prevotella sp900314945 | 1.087 | 0.846 | 0.451 | 0.361 |  |  | 1 |
| 261 | 17.24 | 1.72 | g__Prevotella | s__Prevotella sp900315035 | 0.053 | 0.000 | 0.035 | 6.855 | 1 |  |  |
| 521 | 90.87 | 25.49 | g__Prevotella | s__Prevotella sp900315095 | 0.262 | 0.018 | 0.001 | 3.899 | 1 |  |  |
| 913 | 20.69 | 0 | g__Prevotella | s__Prevotella sp900315545 | 0.010 | 0.012 | 0.004 | -0.290 |  | 1 |  |
| 127 | 10.34 | 0 | g__Prevotella | s__Prevotella sp900315545 | 0.003 | 0.003 | 0.003 | -0.322 |  | 1 |  |
| 899 | 97.48 | 1.01 | g__Prevotella | s__Prevotella sp900315635 | 0.212 | 2.701 | 0.001 | -3.674 |  | 1 |  |
| 312 | 35.17 | 0 | g__Prevotella | s__Prevotella sp900315775 | 0.032 | 0.005 | 0.260 | 2.631 |  |  | 1 |
| 559 | 81.64 | 3.02 | g__Prevotella | s__Prevotella sp900315835 | 0.055 | 0.704 | 0.005 | -3.677 |  | 1 |  |
| 217 | 29.31 | 0 | g__Prevotella | s__Prevotella sp900315955 | 0.005 | 0.076 | 0.076 | -3.906 |  |  | 1 |
| 696 | 97.41 | 44.67 | g__Prevotella | s__Prevotella sp900316285 | 1.004 | 0.033 | 0.001 | 4.948 | 1 |  |  |
| 264 | 93.02 | 11.42 | g__Prevotella | s__Prevotella sp900316295 | 0.450 | 0.008 | 0.012 | 5.832 | 1 |  |  |
| 574 | 95.96 | 29.2 | g__Prevotella | s__Prevotella sp900316475 | 0.917 | 0.480 | 0.756 | 0.932 |  |  | 1 |
| 874 | 89.66 | 11.32 | g__Prevotella | s__Prevotella sp900316565 | 0.389 | 0.968 | 0.043 | -1.315 |  | 1 |  |
| 778 | 35.85 | 1.6 | g__Prevotella | s__Prevotella sp900316645 | 0.465 | 0.005 | 0.070 | 6.615 |  |  | 1 |
| 482 | 67.87 | 55.17 | g__Prevotella | s__Prevotella sp900318395 | 1.376 | 0.008 | 0.005 | 7.407 | 1 |  |  |
| 262 | 74.14 | 8.62 | g__Prevotella | s__Prevotella sp900318625 | 0.534 | 0.014 | 0.009 | 5.269 | 1 |  |  |
| 675 | 73.75 | 22.57 | g__Prevotella | s__Prevotella sp900318795 | 1.373 | 0.027 | 0.116 | 5.688 |  |  | 1 |
| 290 | 88.01 | 2.65 | g__Prevotella | s__Prevotella sp900318855 | 0.013 | 0.427 | 0.002 | -5.046 |  | 1 |  |
| 545 | 50.00 | 3.45 | g__Prevotella | s__Prevotella sp900318915 | 0.132 | 0.005 | 0.000 | 4.627 | 1 |  |  |
| 391 | 27.05 | 1.72 | g__Prevotella | s__Prevotella sp900319715 | 0.183 | 0.001 | 0.000 | 7.513 | 1 |  |  |
| 457 | 89.92 | 8.9 | g__Prevotella | s__Prevotella sp900319905 | 0.562 | 5.935 | 0.007 | -3.400 |  | 1 |  |
| 742 | 86.71 | 2.5 | g__Prevotella | s__Prevotella sp900322095 | 0.051 | 2.834 | 0.004 | -5.805 |  | 1 |  |
| 781 | 66.64 | 35.61 | g__RC9 | s__ | 0.751 | 0.003 | 0.000 | 8.025 | 1 |  |  |
| 718 | 30.11 | 2.7 | g__RC9 | s__ | 0.212 | 0.003 | 0.000 | 6.262 | 1 |  |  |
| 756 | 54.54 | 6.9 | g__RC9 | s__ | 0.161 | 0.001 | 0.002 | 7.039 | 1 |  |  |
| 820 | 26.65 | 6.9 | g__RC9 | s__ | 0.105 | 0.001 | 0.000 | 7.050 | 1 |  |  |
| 447 | 64.80 | 22.3 | g__RC9 | s__ | 0.096 | 0.005 | 0.019 | 4.172 | 1 |  |  |
| 611 | 23.35 | 0 | g__RC9 | s__ | 0.044 | 0.000 | 0.000 | 6.672 | 1 |  |  |
| 192 | 89.21 | 4.31 | g__RC9 | s__ | 0.031 | 0.187 | 0.007 | -2.602 |  | 1 |  |
| 712 | 97.38 | 7.14 | g__RC9 | s__RC9 sp900316045 | 0.054 | 0.284 | 0.013 | -2.407 |  | 1 |  |
| 487 | 39.99 | 2.65 | g__RC9 | s__RC9 sp900317925 | 0.214 | 0.007 | 0.001 | 4.965 | 1 |  |  |
| 412 | 30.88 | 5.26 | g__RC9 | s__RC9 sp900320185 | 0.050 | 0.000 | 0.006 | 6.771 | 1 |  |  |
| 489 | 42.24 | 3.45 | g__RF16 | s__ | 0.019 | 0.000 | 0.009 | 7.427 | 1 |  |  |
| 760 | 58.17 | 22.58 | g__UBA1711 | s__ | 0.204 | 0.013 | 0.000 | 3.921 | 1 |  |  |
| 351 | 50.70 | 13.96 | g__UBA1711 | s__ | 0.131 | 0.002 | 0.000 | 5.908 | 1 |  |  |
| 736 | 33.62 | 0 | g__UBA1711 | s__ | 0.080 | 0.001 | 0.001 | 5.828 | 1 |  |  |
| 219 | 47.77 | 3.22 | g__UBA2918 | s__ | 0.030 | 0.010 | 0.620 | 1.521 |  |  | 1 |
| 848 | 56.72 | 2.39 | g__UBA3839 | s__ | 0.111 | 0.038 | 0.729 | 1.526 |  |  | 1 |
| 880 | 31.90 | 3.45 | g__UBA3839 | s__ | 0.058 | 0.003 | 0.000 | 4.520 | 1 |  |  |
| 197 | 39.83 | 0 | g__UBA4334 | s__ | 0.063 | 0.036 | 0.658 | 0.823 |  |  | 1 |
| 68 | 55.37 | 2.44 | g__UBA4334 | s__ | 0.053 | 0.001 | 0.057 | 6.104 |  |  | 1 |
| 287 | 74.14 | 18.1 | g__UBA4334 | s__UBA4334 sp900316505 | 0.338 | 0.035 | 0.006 | 3.254 | 1 |  |  |
| 550 | 41.38 | 1.72 | g__UBA4372 | s__ | 0.074 | 0.002 | 0.046 | 5.404 | 1 |  |  |
| 879 | 72.34 | 5.17 | g__UBA4372 | s__ | 0.067 | 0.012 | 0.031 | 2.471 | 1 |  |  |
| 315 | 25.71 | 8.62 | g__UBA4372 | s__ | 0.034 | 0.001 | 0.000 | 5.214 | 1 |  |  |
| 693 | 29.31 | 0 | g__UBA6398 | s__UBA6398 sp900314335 | 0.004 | 0.084 | 0.001 | -4.380 |  | 1 |  |
| 583 | 17.95 | 0 | g__UBA6398 | s__UBA6398 sp900314335 | 0.003 | 0.073 | 0.001 | -4.720 |  | 1 |  |
| 690 | 13.43 | 0 | g__UBA6398 | s__UBA6398 sp900314335 | 0.002 | 0.057 | 0.000 | -5.171 |  | 1 |  |
| 322 | 24.55 | 2.73 | g__ | s__ | 0.034 | 0.012 | 0.676 | 1.495 |  |  | 1 |
| 860 | 35.34 | 1.72 | g__CAG-484 | s__ | 0.006 | 0.002 | 0.697 | 1.481 |  |  | 1 |
| 473 | 37.77 | 12.73 | g__UBA2813 | s__ | 0.015 | 0.170 | 0.085 | -3.518 |  |  | 1 |
| 135 | 39.32 | 2.56 | g__UBA2813 | s__UBA2813 sp900319365 | 0.001 | 0.201 | 0.078 | -7.437 |  |  | 1 |
| 631 | 20.94 | 0 | g__UBA2813 | s__UBA2813 sp900319365 | 0.009 | 0.083 | 0.079 | -3.234 |  |  | 1 |
| 345 | 38.27 | 2.56 | g__Zag111 | s__ | 0.009 | 0.001 | 0.044 | 3.964 | 1 |  |  |
| 645 | 89.20 | 3.16 | g__Desulfovibrio | s__Desulfovibrio sp900319575 | 0.024 | 0.149 | 0.000 | -2.652 |  | 1 |  |
| 82 | 21.16 | 0 | g__Fibrobacter | s__ | 0.042 | 0.003 | 0.001 | 3.730 | 1 |  |  |
| 616 | 25.86 | 0 | g__Fibrobacter | s__ | 0.109 | 0.003 | 0.000 | 5.142 | 1 |  |  |
| 758 | 27.59 | 0 | g__Fibrobacter | s__ | 0.098 | 0.004 | 0.002 | 4.744 | 1 |  |  |
| 810 | 13.20 | 0 | g__Fibrobacter_A | s__Fibrobacter_A sp002390045 | 0.017 | 0.008 | 0.942 | 1.132 |  |  | 1 |
| 554 | 46.61 | 35.12 | g__ | s__ | 0.661 | 0.027 | 0.000 | 4.622 | 1 |  |  |
| 632 | 47.05 | 11.56 | g__ | s__ | 0.003 | 0.001 | 0.110 | 2.240 |  |  | 1 |
| 634 | 31.58 | 1.75 | g__ | s__ | 0.005 | 0.014 | 0.170 | -1.393 |  |  | 1 |
| 765 | 82.46 | 29.59 | g__ | s__ | 0.002 | 0.022 | 0.100 | -3.293 |  |  | 1 |
| 21 | 68.87 | 0 | g__CAG-536 | s__ | 0.008 | 0.007 | 0.205 | 0.316 |  |  | 1 |
| 13 | 25.04 | 0 | g__CAG-536 | s__CAG-536 sp002394635 | 0.018 | 0.005 | 0.326 | 1.864 |  |  | 1 |
| 41 | 91.37 | 0 | g__CAG-536 | s__CAG-536 sp900314505 | 0.033 | 0.052 | 0.107 | -0.660 |  |  | 1 |
| 340 | 99.06 | 21.23 | g__Kandleria | s__Kandleria sp900317015 | 0.042 | 0.566 | 0.044 | -3.762 |  | 1 |  |
| 145 | 96.23 | 6.06 | g__Kandleria | s__Kandleria sp900317725 | 0.018 | 0.230 | 0.055 | -3.645 |  |  | 1 |
| 639 | 99.06 | 7.55 | g__Sharpea | s__Sharpea azabuensis | 0.085 | 0.131 | 0.077 | -0.623 |  |  | 1 |
| 587 | 84.50 | 6.35 | g__Solobacterium | s__ | 0.196 | 0.012 | 0.005 | 4.066 | 1 |  |  |
| 503 | 24.14 | 0 | g__Solobacterium | s__ | 0.016 | 0.000 | 0.000 | 5.353 | 1 |  |  |
| 80 | 12.07 | 0 | g__Solobacterium | s__ | 0.016 | 0.000 | 0.001 | 6.203 | 1 |  |  |
| 469 | 98.27 | 1.27 | g__Solobacterium | s__Solobacterium sp900290205 | 0.004 | 0.351 | 0.003 | -6.446 |  | 1 |  |
| 231 | 96.55 | 4.04 | g__Solobacterium | s__Solobacterium sp900314345 | 0.108 | 4.090 | 0.006 | -5.245 |  | 1 |  |
| 534 | 90.48 | 1.75 | g__Solobacterium | s__Solobacterium sp900315065 | 0.008 | 0.283 | 0.049 | -5.101 |  | 1 |  |
| 298 | 86.35 | 0 | g__Solobacterium | s__Solobacterium sp900319505 | 0.020 | 0.169 | 0.126 | -3.089 |  |  | 1 |
| 479 | 22.74 | 0 | g__Solobacterium | s__Solobacterium sp900343155 | 0.000 | 0.012 | 0.037 | -4.835 |  | 1 |  |
| 579 | 43.17 | 0.95 | g__Solobacterium | s__Solobacterium timonensis | 0.002 | 0.046 | 0.047 | -4.522 |  | 1 |  |
| 619 | 69.38 | 8.8 | g__UBA11963 | s__ | 0.014 | 0.001 | 0.016 | 4.686 | 1 |  |  |
| 147 | 59.44 | 2.25 | g__UBA2730 | s__UBA2730 sp900313345 | 0.001 | 0.005 | 0.048 | -2.877 |  | 1 |  |
| 635 | 91.57 | 2.41 | g__UBA2730 | s__UBA2730 sp900319955 | 0.002 | 0.032 | 0.064 | -4.307 |  |  | 1 |
| 188 | 25.98 | 1.12 | g__UBA2730 | s__UBA2730 sp900320505 | 0.000 | 0.004 | 0.017 | -4.980 |  | 1 |  |
| 529 | 79.21 | 13.37 | g__UBA3789 | s__ | 0.003 | 0.110 | 0.014 | -5.075 |  | 1 |  |
| 633 | 81.33 | 31.56 | g__UBA3789 | s__UBA3789 sp900314225 | 0.002 | 0.099 | 0.241 | -5.661 |  |  | 1 |
| 466 | 17.54 | 0 | g__UBA4951 | s__ | 0.012 | 0.000 | 0.001 | 4.966 | 1 |  |  |
| 543 | 85.67 | 3.97 | g__UBA636 | s__UBA636 sp900321955 | 0.100 | 0.001 | 0.001 | 7.181 | 1 |  |  |
| 789 | 83.76 | 2.36 | g__UBA733 | s__UBA733 sp900316365 | 0.082 | 0.023 | 0.132 | 1.814 |  |  | 1 |
| 165 | 25.17 | 2.25 | g__UBA7642 | s__ | 0.005 | 0.001 | 0.014 | 3.052 | 1 |  |  |
| 731 | 86.54 | 2.25 | g__UBA7642 | s__UBA7642 sp900316035 | 0.003 | 0.043 | 0.090 | -3.939 |  |  | 1 |
| 98 | 28.48 | 0.67 | g__ | s__ | 0.003 | 0.003 | 0.136 | 0.007 |  |  | 1 |
| 646 | 100.00 | 229.21 | g__ | s__ | 0.005 | 0.059 | 0.048 | -3.695 |  | 1 |  |
| 235 | 60.58 | 4.35 | g__ | s__ | 0.000 | 0.013 | 0.345 | -5.124 |  |  | 1 |
| 123 | 38.85 | 0.48 | g__ | s__ | 0.004 | 0.009 | 0.049 | -1.189 |  | 1 |  |
| 381 | 31.30 | 3.55 | g__ | s__ | 0.107 | 0.049 | 0.946 | 1.135 |  |  | 1 |
| 398 | 35.13 | 3.76 | g__ | s__ | 0.052 | 0.002 | 0.113 | 4.605 |  |  | 1 |
| 357 | 67.90 | 1.68 | g__ | s__ | 0.202 | 0.001 | 0.001 | 7.363 | 1 |  |  |
| 472 | 32.97 | 0 | g__ | s__ | 0.001 | 0.002 | 0.061 | -1.445 |  |  | 1 |
| 16 | 95.74 | 0.71 | g__ | s__ | 0.164 | 0.036 | 0.039 | 2.209 | 1 |  |  |
| 490 | 58.25 | 0.85 | g__ | s__ | 0.013 | 0.012 | 0.277 | 0.092 |  |  | 1 |
| 527 | 83.40 | 5.65 | g__ | s__ | 0.245 | 0.056 | 0.140 | 2.115 |  |  | 1 |
| 462 | 43.57 | 2.21 | g__ | s__ | 0.135 | 0.001 | 0.001 | 7.984 | 1 |  |  |
| 537 | 93.55 | 2.07 | g__ | s__ | 0.111 | 0.115 | 0.274 | -0.055 |  |  | 1 |
| 626 | 73.23 | 1.4 | g__ | s__ | 0.111 | 0.001 | 0.055 | 7.672 |  |  | 1 |
| 819 | 19.24 | 0.4 | g__ | s__ | 0.036 | 0.000 | 0.000 | 7.046 | 1 |  |  |
| 644 | 20.80 | 1.89 | g__ | s__ | 0.026 | 0.000 | 0.003 | 6.567 | 1 |  |  |
| 799 | 72.81 | 3.16 | g__ | s__ | 0.004 | 0.123 | 0.090 | -5.052 |  |  | 1 |
| 289 | 11.96 | 4.11 | g__ | s__ | 0.014 | 0.000 | 0.000 | 5.973 | 1 |  |  |
| 308 | 20.38 | 0 | g__ | s__ | 0.010 | 0.001 | 0.002 | 3.747 | 1 |  |  |
| 477 | 53.48 | 8.02 | g__Blautia_A | s__ | 0.074 | 0.001 | 0.001 | 5.946 | 1 |  |  |
| 278 | 32.88 | 0 | g__Butyrivibrio | s__Butyrivibrio fibrisolvens_C | 0.014 | 0.001 | 0.005 | 4.535 | 1 |  |  |
| 248 | 64.39 | 24 | g__CAG-103 | s__ | 0.234 | 0.002 | 0.002 | 6.869 | 1 |  |  |
| 173 | 15.52 | 1.72 | g__CAG-103 | s__ | 0.015 | 0.000 | 0.003 | 7.349 | 1 |  |  |
| 348 | 22.41 | 0 | g__CAG-103 | s__ | 0.007 | 0.000 | 0.001 | 4.318 | 1 |  |  |
| 593 | 37.93 | 0 | g__CAG-103 | s__CAG-103 sp900316745 | 0.034 | 0.003 | 0.007 | 3.532 | 1 |  |  |
| 491 | 77.59 | 7.08 | g__CAG-103 | s__CAG-103 sp900317855 | 0.025 | 0.333 | 0.274 | -3.737 |  |  | 1 |
| 729 | 18.97 | 0 | g__CAG-110 | s__ | 0.086 | 0.000 | 0.038 | 7.939 | 1 |  |  |
| 286 | 84.56 | 1.34 | g__CAG-127 | s__CAG-127 sp900319515 | 0.006 | 0.163 | 0.018 | -4.854 |  | 1 |  |
| 326 | 45.79 | 10.17 | g__CAG-269 | s__ | 0.002 | 0.014 | 0.085 | -2.941 |  |  | 1 |
| 396 | 69.61 | 18.01 | g__CAG-269 | s__CAG-269 sp001916005 | 0.004 | 0.019 | 0.048 | -2.371 |  | 1 |  |
| 341 | 98.18 | 124.93 | g__CAG-269 | s__CAG-269 sp002372935 | 0.007 | 0.021 | 0.054 | -1.608 |  |  | 1 |
| 116 | 38.99 | 0 | g__CAG-411 | s__ | 0.020 | 0.017 | 0.545 | 0.224 |  |  | 1 |
| 551 | 81.75 | 17.54 | g__CAG-465 | s__ | 0.001 | 0.011 | 0.241 | -3.731 |  |  | 1 |
| 927 | 70.57 | 1.05 | g__CAG-465 | s__ | 0.010 | 0.035 | 0.111 | -1.785 |  |  | 1 |
| 801 | 30.36 | 3.48 | g__CAG-590 | s__ | 0.019 | 0.001 | 0.005 | 5.033 | 1 |  |  |
| 346 | 87.72 | 10.73 | g__CAG-603 | s__CAG-603 sp900314525 | 0.079 | 0.044 | 0.826 | 0.848 |  |  | 1 |
| 832 | 97.23 | 4.72 | g__CAG-603 | s__CAG-603 sp900315425 | 0.051 | 0.002 | 0.001 | 4.799 | 1 |  |  |
| 909 | 92.82 | 5.3 | g__CAG-603 | s__CAG-603 sp900321855 | 0.132 | 0.013 | 0.140 | 3.347 |  |  | 1 |
| 377 | 89.80 | 5.29 | g__CAG-791 | s__ | 0.028 | 0.500 | 0.012 | -4.181 |  | 1 |  |
| 504 | 40.51 | 5.01 | g__CAG-791 | s__ | 0.077 | 0.485 | 0.026 | -2.661 |  | 1 |  |
| 100 | 17.54 | 0 | g__CAG-791 | s__ | 0.041 | 0.007 | 0.100 | 2.601 |  |  | 1 |
| 148 | 86.79 | 19.96 | g__CAG-791 | s__ | 0.383 | 0.291 | 0.438 | 0.398 |  |  | 1 |
| 285 | 88.22 | 6.65 | g__CAG-791 | s__ | 0.443 | 0.002 | 0.000 | 7.519 | 1 |  |  |
| 921 | 75.44 | 22.46 | g__CAG-791 | s__ | 0.338 | 0.015 | 0.000 | 4.485 | 1 |  |  |
| 375 | 71.76 | 19.73 | g__CAG-791 | s__ | 0.258 | 0.007 | 0.000 | 5.176 | 1 |  |  |
| 533 | 51.20 | 21.05 | g__CAG-791 | s__ | 0.245 | 0.016 | 0.022 | 3.947 | 1 |  |  |
| 779 | 60.38 | 0.86 | g__CAG-791 | s__ | 0.232 | 0.006 | 0.005 | 5.201 | 1 |  |  |
| 808 | 31.66 | 0 | g__CAG-791 | s__ | 0.189 | 0.005 | 0.001 | 5.368 | 1 |  |  |
| 614 | 60.53 | 8.77 | g__CAG-791 | s__ | 0.171 | 0.005 | 0.000 | 4.971 | 1 |  |  |
| 605 | 82.53 | 30.64 | g__CAG-791 | s__ | 0.439 | 0.134 | 0.508 | 1.713 |  |  | 1 |
| 63 | 54.39 | 8.77 | g__CAG-791 | s__ | 0.170 | 0.015 | 0.008 | 3.540 | 1 |  |  |
| 855 | 30.70 | 8.77 | g__CAG-791 | s__ | 0.167 | 0.027 | 0.030 | 2.631 | 1 |  |  |
| 905 | 54.23 | 30.06 | g__CAG-791 | s__ | 0.141 | 0.010 | 0.002 | 3.845 | 1 |  |  |
| 296 | 52.37 | 5.39 | g__CAG-791 | s__ | 0.103 | 0.001 | 0.001 | 6.236 | 1 |  |  |
| 570 | 15.79 | 0 | g__CAG-791 | s__ | 0.010 | 0.003 | 0.505 | 1.812 |  |  | 1 |
| 140 | 32.76 | 6.9 | g__CAG-791 | s__ | 0.086 | 0.005 | 0.000 | 4.132 | 1 |  |  |
| 344 | 49.12 | 5.26 | g__CAG-791 | s__ | 0.080 | 0.001 | 0.001 | 6.516 | 1 |  |  |
| 782 | 25.53 | 1.72 | g__CAG-791 | s__ | 0.075 | 0.001 | 0.000 | 6.900 | 1 |  |  |
| 926 | 25.44 | 7.02 | g__CAG-791 | s__ | 0.056 | 0.007 | 0.001 | 3.104 | 1 |  |  |
| 236 | 15.79 | 0 | g__CAG-791 | s__ | 0.030 | 0.000 | 0.000 | 7.965 | 1 |  |  |
| 274 | 31.05 | 0.89 | g__CAG-791 | s__CAG-791 sp002449595 | 0.043 | 0.001 | 0.000 | 6.201 | 1 |  |  |
| 113 | 80.70 | 13.16 | g__CAG-791 | s__CAG-791 sp900101015 | 0.552 | 0.009 | 0.000 | 5.945 | 1 |  |  |
| 464 | 95.11 | 3.42 | g__CAG-791 | s__CAG-791 sp900315055 | 0.013 | 2.589 | 0.198 | -7.621 |  |  | 1 |
| 650 | 75.31 | 1.87 | g__CAG-791 | s__CAG-791 sp900316855 | 0.049 | 0.087 | 0.264 | -0.836 |  |  | 1 |
| 52 | 93.85 | 1.26 | g__CAG-791 | s__CAG-791 sp900317475 | 0.119 | 0.764 | 0.031 | -2.685 |  | 1 |  |
| 655 | 92.82 | 0.19 | g__CAG-791 | s__CAG-791 sp900317555 | 0.425 | 0.683 | 0.002 | -0.684 |  | 1 |  |
| 474 | 47.37 | 10.53 | g__CAG-791 | s__CAG-791 sp900318875 | 0.074 | 0.769 | 0.034 | -3.379 |  | 1 |  |
| 382 | 95.25 | 49.07 | g__CAG-791 | s__CAG-791 sp900321785 | 0.755 | 0.478 | 0.664 | 0.661 |  |  | 1 |
| 119 | 20.69 | 0 | g__CAG-793 | s__ | 0.000 | 0.012 | 0.170 | -4.872 |  |  | 1 |
| 715 | 96.49 | 138.04 | g__CAG-793 | s__ | 0.010 | 0.136 | 0.009 | -3.774 |  | 1 |  |
| 146 | 47.85 | 0.67 | g__CAG-793 | s__ | 0.000 | 0.015 | 0.088 | -5.031 |  |  | 1 |
| 159 | 34.72 | 3.36 | g__CAG-793 | s__ | 0.004 | 0.000 | 0.064 | 5.345 |  |  | 1 |
| 431 | 66.88 | 4.28 | g__CAG-793 | s__ | 0.004 | 0.092 | 0.103 | -4.383 |  |  | 1 |
| 698 | 94.74 | 11.16 | g__CAG-793 | s__ | 0.002 | 0.075 | 0.083 | -5.474 |  |  | 1 |
| 713 | 69.10 | 1.79 | g__CAG-793 | s__ | 0.012 | 0.000 | 0.003 | 4.913 | 1 |  |  |
| 76 | 32.73 | 0 | g__CAG-793 | s__ | 0.001 | 0.000 | 0.016 | 4.861 | 1 |  |  |
| 571 | 81.12 | 2.28 | g__Clostridium | s__ | 0.010 | 0.075 | 0.206 | -2.886 |  |  | 1 |
| 721 | 14.04 | 0 | g__Clostridium_R | s__ | 0.011 | 0.151 | 0.004 | -3.744 |  | 1 |  |
| 72 | 87.34 | 4 | g__Clostridium_R | s__Clostridium_R sp003445895 | 0.257 | 0.038 | 0.001 | 2.754 | 1 |  |  |
| 361 | 81.65 | 0 | g__Clostridium_R | s__Clostridium_R sp900321355 | 0.005 | 0.519 | 0.003 | -6.596 |  | 1 |  |
| 592 | 55.45 | 0 | g__Eubacterium_A | s__ | 0.001 | 0.023 | 0.000 | -4.901 |  | 1 |  |
| 342 | 63.12 | 1.42 | g__Eubacterium_A | s__ | 0.004 | 0.035 | 0.084 | -3.295 |  |  | 1 |
| 484 | 41.31 | 0.71 | g__Eubacterium_A | s__ | 0.021 | 0.000 | 0.010 | 6.459 | 1 |  |  |
| 556 | 92.55 | 1.77 | g__Eubacterium_A | s__Eubacterium_A pyruvativorans | 0.096 | 0.316 | 0.024 | -1.725 |  | 1 |  |
| 923 | 96.39 | 4.08 | g__Eubacterium_A | s__Eubacterium_A sp000688015 | 0.134 | 0.066 | 0.799 | 1.023 |  |  | 1 |
| 89 | 15.17 | 0 | g__Eubacterium_A | s__Eubacterium_A sp900318155 | 0.007 | 0.003 | 0.735 | 0.953 |  |  | 1 |
| 720 | 22.81 | 0.35 | g__Eubacterium_E | s__ | 0.012 | 0.000 | 0.047 | 5.209 | 1 |  |  |
| 914 | 100.00 | 3.01 | g__Eubacterium_E | s__ | 0.009 | 0.271 | 0.272 | -4.915 |  |  | 1 |
| 694 | 13.50 | 0 | g__Eubacterium_F | s__ | 0.002 | 0.015 | 0.069 | -2.950 |  |  | 1 |
| 563 | 47.41 | 7.86 | g__Eubacterium_H | s__ | 0.023 | 0.466 | 0.005 | -4.322 |  | 1 |  |
| 797 | 40.35 | 1.75 | g__Eubacterium_H | s__ | 0.029 | 0.427 | 0.019 | -3.894 |  | 1 |  |
| 275 | 56.90 | 0 | g__Eubacterium_H | s__ | 0.014 | 0.259 | 0.017 | -4.164 |  | 1 |  |
| 301 | 50.32 | 1.59 | g__Eubacterium_H | s__ | 0.037 | 0.160 | 0.020 | -2.116 |  | 1 |  |
| 854 | 14.01 | 1.91 | g__Eubacterium_H | s__ | 0.006 | 0.074 | 0.000 | -3.721 |  | 1 |  |
| 124 | 12.28 | 0 | g__Eubacterium_H | s__ | 0.001 | 0.051 | 0.047 | -5.174 |  | 1 |  |
| 434 | 82.51 | 0.64 | g__Eubacterium_H | s__ | 0.005 | 0.062 | 0.193 | -3.573 |  |  | 1 |
| 834 | 10.53 | 0 | g__Eubacterium_H | s__ | 0.004 | 0.020 | 0.115 | -2.318 |  |  | 1 |
| 542 | 67.62 | 1.91 | g__Eubacterium_H | s__Eubacterium_H cellulosolvens | 0.057 | 0.098 | 0.134 | -0.775 |  |  | 1 |
| 586 | 19.15 | 0.64 | g__Eubacterium_H | s__Eubacterium_H sp002368795 | 0.009 | 0.000 | 0.003 | 5.168 | 1 |  |  |
| 488 | 96.58 | 0 | g__Eubacterium_H | s__Eubacterium_H sp900318405 | 0.081 | 0.223 | 0.159 | -1.458 |  |  | 1 |
| 812 | 95.22 | 3.5 | g__Eubacterium_H | s__Eubacterium_H sp900319355 | 0.086 | 0.722 | 0.044 | -3.076 |  | 1 |  |
| 199 | 33.10 | 1.29 | g__Eubacterium_I | s__ | 0.008 | 0.004 | 0.700 | 0.850 |  |  | 1 |
| 368 | 15.79 | 1.75 | g__Eubacterium_Q | s__ | 0.005 | 0.067 | 0.038 | -3.621 |  | 1 |  |
| 421 | 19.30 | 0 | g__Eubacterium_Q | s__ | 0.008 | 0.001 | 0.071 | 2.859 |  |  | 1 |
| 744 | 61.40 | 13.16 | g__Eubacterium_Q | s__ | 0.046 | 0.002 | 0.021 | 4.382 | 1 |  |  |
| 411 | 41.23 | 5.26 | g__Eubacterium_Q | s__ | 0.044 | 0.002 | 0.002 | 4.279 | 1 |  |  |
| 780 | 31.02 | 1.75 | g__Eubacterium_Q | s__Eubacterium_Q sp000687655 | 0.015 | 0.016 | 0.445 | -0.065 |  |  | 1 |
| 208 | 28.36 | 0 | g__Eubacterium_Q | s__Eubacterium_Q sp002350525 | 0.014 | 0.001 | 0.002 | 4.563 | 1 |  |  |
| 238 | 91.48 | 4.82 | g__Eubacterium_Q | s__Eubacterium_Q sp002368685 | 0.052 | 0.062 | 0.138 | -0.237 |  |  | 1 |
| 343 | 81.21 | 2.1 | g__Eubacterium_Q | s__Eubacterium_Q sp900314445 | 0.052 | 0.271 | 0.075 | -2.371 |  |  | 1 |
| 547 | 24.82 | 0.71 | g__Firm-16 | s__Firm-16 sp002368805 | 0.037 | 0.000 | 0.007 | 7.602 | 1 |  |  |
| 684 | 45.09 | 0 | g__GCA-900066995 | s__ | 0.003 | 0.009 | 0.193 | -1.795 |  |  | 1 |
| 440 | 86.24 | 10.29 | g__GCA-900199385 | s__ | 0.635 | 0.002 | 0.006 | 8.520 | 1 |  |  |
| 911 | 38.86 | 0.81 | g__GCA-900199385 | s__ | 0.178 | 0.001 | 0.043 | 8.210 | 1 |  |  |
| 869 | 27.59 | 9.48 | g__GCA-900199385 | s__ | 0.058 | 0.001 | 0.002 | 6.462 | 1 |  |  |
| 580 | 31.03 | 0 | g__GCA-900199385 | s__ | 0.026 | 0.000 | 0.046 | 8.434 | 1 |  |  |
| 618 | 65.52 | 12.07 | g__GCA-900199385 | s__GCA-900199385 sp900320755 | 0.477 | 0.001 | 0.009 | 8.841 | 1 |  |  |
| 365 | 54.39 | 0 | g__Lachnospira | s__ | 0.002 | 0.033 | 0.109 | -3.909 |  |  | 1 |
| 764 | 93.06 | 3.83 | g__Lachnospira | s__Lachnospira multipara | 0.011 | 0.033 | 0.253 | -1.591 |  |  | 1 |
| 228 | 50.18 | 3.51 | g__Lachnospira | s__Lachnospira sp000437735 | 0.007 | 0.007 | 0.089 | 0.013 |  |  | 1 |
| 847 | 92.11 | 4.97 | g__Lachnospira | s__Lachnospira sp003537285 | 0.025 | 0.367 | 0.217 | -3.884 |  |  | 1 |
| 24 | 96.55 | 52.66 | g__Mogibacterium | s__ | 0.207 | 0.070 | 0.676 | 1.559 |  |  | 1 |
| 99 | 58.51 | 0 | g__Mogibacterium | s__ | 0.010 | 0.074 | 0.172 | -2.877 |  |  | 1 |
| 138 | 24.14 | 0 | g__Mogibacterium | s__ | 0.002 | 0.013 | 0.270 | -2.921 |  |  | 1 |
| 213 | 55.80 | 2.35 | g__Mogibacterium | s__ | 0.007 | 0.008 | 0.045 | -0.190 |  | 1 |  |
| 465 | 85.23 | 30.57 | g__Mogibacterium | s__ | 0.198 | 0.005 | 0.014 | 5.311 | 1 |  |  |
| 162 | 13.79 | 5.17 | g__Mogibacterium | s__ | 0.005 | 0.000 | 0.000 | 5.280 | 1 |  |  |
| 393 | 68.07 | 0.35 | g__Mogibacterium | s__Mogibacterium sp900315625 | 0.002 | 0.033 | 0.004 | -3.978 |  | 1 |  |
| 241 | 20.90 | 0.05 | g__NK4A136 | s__NK4A136 sp000687675 | 0.008 | 0.000 | 0.010 | 4.176 | 1 |  |  |
| 795 | 29.66 | 0.48 | g__NK4A144 | s__ | 0.030 | 0.000 | 0.001 | 6.831 | 1 |  |  |
| 191 | 25.94 | 0 | g__NK4A144 | s__ | 0.023 | 0.000 | 0.010 | 7.323 | 1 |  |  |
| 685 | 27.59 | 0 | g__Oribacterium | s__ | 0.037 | 0.065 | 0.125 | -0.825 |  |  | 1 |
| 383 | 77.00 | 0 | g__Oribacterium | s__Oribacterium sp900315665 | 0.317 | 0.423 | 0.201 | -0.417 |  |  | 1 |
| 115 | 18.31 | 0.7 | g__Pseudoramibacter | s__ | 0.002 | 0.102 | 0.001 | -5.777 |  | 1 |  |
| 102 | 10.34 | 0 | g__Pseudoramibacter | s__ | 0.001 | 0.045 | 0.001 | -5.251 |  | 1 |  |
| 850 | 36.21 | 1.72 | g__Pseudoramibacter | s__ | 0.000 | 0.023 | 0.004 | -5.848 |  | 1 |  |
| 130 | 25.00 | 0 | g__Pseudoramibacter | s__ | 0.000 | 0.013 | 0.005 | -5.975 |  | 1 |  |
| 562 | 40.52 | 1.72 | g__RUG100 | s__ | 0.005 | 0.030 | 0.116 | -2.585 |  |  | 1 |
| 904 | 16.11 | 0 | g__RUG147 | s__RUG147 sp900315495 | 0.001 | 0.017 | 0.052 | -4.909 |  |  | 1 |
| 461 | 28.87 | 0 | g__RUG306 | s__ | 0.036 | 0.027 | 0.667 | 0.425 |  |  | 1 |
| 257 | 65.39 | 1.69 | g__RUG306 | s__ | 0.071 | 0.001 | 0.006 | 6.107 | 1 |  |  |
| 763 | 90.43 | 25.18 | g__RUG306 | s__ | 0.089 | 2.331 | 0.278 | -4.715 |  |  | 1 |
| 623 | 58.88 | 0.24 | g__RUG306 | s__RUG306 sp900314085 | 0.069 | 0.013 | 0.573 | 2.463 |  |  | 1 |
| 335 | 22.81 | 0 | g__RUG306 | s__RUG306 sp900316075 | 0.064 | 0.181 | 0.130 | -1.501 |  |  | 1 |
| 867 | 94.09 | 0.76 | g__RUG306 | s__RUG306 sp900317755 | 0.023 | 0.358 | 0.097 | -3.970 |  |  | 1 |
| 467 | 97.20 | 0.67 | g__RUG420 | s__ | 0.006 | 0.863 | 0.000 | -7.077 |  | 1 |  |
| 680 | 84.56 | 3.69 | g__RUG420 | s__ | 0.851 | 0.040 | 0.000 | 4.419 | 1 |  |  |
| 608 | 23.51 | 0 | g__RUG472 | s__ | 0.022 | 0.000 | 0.012 | 6.847 | 1 |  |  |
| 871 | 91.91 | 136.7 | g__RUG740 | s__ | 0.425 | 0.002 | 0.004 | 7.794 | 1 |  |  |
| 788 | 64.64 | 8.62 | g__RUG740 | s__ | 0.298 | 0.001 | 0.042 | 8.137 | 1 |  |  |
| 776 | 54.59 | 2.68 | g__RUG740 | s__ | 0.150 | 0.001 | 0.013 | 8.103 | 1 |  |  |
| 514 | 33.26 | 4.7 | g__RUG740 | s__ | 0.097 | 0.001 | 0.000 | 7.397 | 1 |  |  |
| 415 | 18.97 | 5.17 | g__RUG740 | s__ | 0.009 | 0.000 | 0.001 | 6.657 | 1 |  |  |
| 700 | 27.59 | 0 | g__RUG740 | s__RUG740 sp900316415 | 0.038 | 0.000 | 0.008 | 7.674 | 1 |  |  |
| 682 | 53.88 | 1.06 | g__RUG754 | s__ | 0.024 | 0.003 | 0.004 | 3.208 | 1 |  |  |
| 163 | 19.47 | 0 | g__RUG754 | s__RUG754 sp900315895 | 0.009 | 0.000 | 0.079 | 4.531 |  |  | 1 |
| 745 | 15.79 | 0 | g__RUG842 | s__ | 0.012 | 0.000 | 0.000 | 6.300 | 1 |  |  |
| 268 | 62.97 | 3.83 | g__Ruminiclostridium_C | s__ | 0.034 | 0.074 | 0.000 | -1.126 |  | 1 |  |
| 10 | 13.79 | 0 | g__Ruminococcus | s__ | 0.007 | 0.115 | 0.005 | -4.127 |  | 1 |  |
| 214 | 17.59 | 0 | g__Ruminococcus | s__ | 0.011 | 0.005 | 0.942 | 1.245 |  |  | 1 |
| 606 | 40.00 | 11.21 | g__Ruminococcus | s__ | 0.048 | 0.005 | 0.003 | 3.308 | 1 |  |  |
| 179 | 41.38 | 5.17 | g__Ruminococcus | s__ | 0.028 | 0.002 | 0.006 | 3.984 | 1 |  |  |
| 882 | 37.33 | 3.02 | g__Ruminococcus | s__ | 0.045 | 0.015 | 0.583 | 1.624 |  |  | 1 |
| 837 | 32.53 | 1.72 | g__Ruminococcus | s__ | 0.008 | 0.000 | 0.003 | 5.434 | 1 |  |  |
| 180 | 82.21 | 1.22 | g__Ruminococcus | s__Ruminococcus sp900319195 | 0.045 | 0.225 | 0.242 | -2.323 |  |  | 1 |
| 29 | 38.60 | 11.4 | g__Ruminococcus_D | s__ | 0.053 | 0.001 | 0.029 | 5.218 | 1 |  |  |
| 617 | 100.00 | 9.09 | g__Ruminococcus_D | s__Ruminococcus_D sp900319075 | 0.106 | 0.037 | 0.715 | 1.525 |  |  | 1 |
| 418 | 56.33 | 3.36 | g__Ruminococcus_E | s__ | 0.074 | 0.000 | 0.089 | 8.660 |  |  | 1 |
| 596 | 75.01 | 15.89 | g__Ruminococcus_E | s__ | 0.080 | 0.001 | 0.072 | 7.211 |  |  | 1 |
| 364 | 83.33 | 1.89 | g__Ruminococcus_E | s__ | 0.063 | 0.000 | 0.045 | 7.609 | 1 |  |  |
| 666 | 63.79 | 3.45 | g__Ruminococcus_E | s__ | 0.346 | 0.001 | 0.071 | 8.789 |  |  | 1 |
| 107 | 42.21 | 1.79 | g__Ruminococcus_E | s__ | 0.034 | 0.000 | 0.025 | 8.138 | 1 |  |  |
| 722 | 20.69 | 2.59 | g__Ruminococcus_E | s__ | 0.031 | 0.000 | 0.013 | 8.130 | 1 |  |  |
| 727 | 56.35 | 19.18 | g__Ruminococcus_E | s__ | 0.041 | 0.000 | 0.074 | 7.647 |  |  | 1 |
| 767 | 92.03 | 8.1 | g__Ruminococcus_E | s__ | 0.128 | 0.001 | 0.182 | 6.985 |  |  | 1 |
| 893 | 15.52 | 1.72 | g__Ruminococcus_E | s__ | 0.020 | 0.000 | 0.027 | 6.184 | 1 |  |  |
| 888 | 98.28 | 22.24 | g__Ruminococcus_E | s__Ruminococcus_E bromii_A | 0.040 | 0.057 | 0.500 | -0.500 |  |  | 1 |
| 824 | 55.17 | 8.62 | g__Ruminococcus_E | s__Ruminococcus_E sp900100595 | 0.117 | 0.000 | 0.016 | 8.354 | 1 |  |  |
| 738 | 48.06 | 0 | g__Ruminococcus_E | s__Ruminococcus_E sp900314705 | 0.027 | 0.014 | 0.915 | 1.010 |  |  | 1 |
| 640 | 63.79 | 3.69 | g__Ruminococcus_E | s__Ruminococcus_E sp900315605 | 0.066 | 0.000 | 0.000 | 7.549 | 1 |  |  |
| 726 | 15.58 | 0.67 | g__Ruminococcus_E | s__Ruminococcus_E sp900316555 | 0.116 | 0.000 | 0.076 | 9.379 |  |  | 1 |
| 240 | 86.97 | 17.36 | g__Ruminococcus_E | s__Ruminococcus_E sp900317315 | 0.439 | 0.001 | 0.047 | 8.214 | 1 |  |  |
| 519 | 84.88 | 9.17 | g__Ruminococcus_E | s__Ruminococcus_E sp900319655 | 0.104 | 0.003 | 0.013 | 5.234 | 1 |  |  |
| 449 | 97.37 | 114.99 | g__Saccharofermentans | s__ | 0.236 | 0.001 | 0.000 | 7.350 | 1 |  |  |
| 62 | 90.38 | 8.56 | g__Saccharofermentans | s__ | 0.209 | 0.001 | 0.000 | 7.790 | 1 |  |  |
| 406 | 15.96 | 0 | g__Saccharofermentans | s__ | 0.040 | 0.000 | 0.001 | 7.905 | 1 |  |  |
| 894 | 35.73 | 8.61 | g__Saccharofermentans | s__ | 0.020 | 0.000 | 0.000 | 6.965 | 1 |  |  |
| 851 | 50.30 | 1.27 | g__Stomatobaculum | s__ | 0.006 | 0.056 | 0.016 | -3.253 |  | 1 |  |
| 318 | 15.79 | 0 | g__Stomatobaculum | s__ | 0.019 | 0.001 | 0.002 | 4.329 | 1 |  |  |
| 409 | 42.03 | 0 | g__TF01-11 | s__ | 0.007 | 0.036 | 0.151 | -2.368 |  |  | 1 |
| 510 | 96.52 | 8.04 | g__UBA1066 | s__ | 0.489 | 0.018 | 0.001 | 4.803 | 1 |  |  |
| 373 | 90.52 | 7.75 | g__UBA1066 | s__ | 0.222 | 0.089 | 0.893 | 1.327 |  |  | 1 |
| 403 | 22.81 | 0 | g__UBA1066 | s__ | 0.042 | 0.001 | 0.001 | 5.914 | 1 |  |  |
| 672 | 83.33 | 0 | g__UBA1066 | s__ | 0.021 | 0.500 | 0.204 | -4.541 |  |  | 1 |
| 371 | 20.44 | 0 | g__UBA1066 | s__ | 0.012 | 0.000 | 0.018 | 6.215 | 1 |  |  |
| 783 | 81.48 | 6.54 | g__UBA1066 | s__UBA1066 sp900314565 | 0.231 | 0.017 | 0.065 | 3.737 |  |  | 1 |
| 501 | 85.28 | 0.64 | g__UBA1066 | s__UBA1066 sp900317045 | 0.007 | 0.320 | 0.105 | -5.437 |  |  | 1 |
| 857 | 97.31 | 8.08 | g__UBA1066 | s__UBA1066 sp900317515 | 0.572 | 0.144 | 0.378 | 1.987 |  |  | 1 |
| 57 | 96.49 | 9.81 | g__UBA1066 | s__UBA1066 sp900319795 | 0.147 | 0.009 | 0.078 | 4.027 |  |  | 1 |
| 775 | 97.45 | 0 | g__UBA1066 | s__UBA1066 sp900320615 | 0.153 | 0.126 | 0.511 | 0.280 |  |  | 1 |
| 111 | 47.37 | 1.75 | g__UBA1081 | s__UBA1081 sp900314355 | 0.001 | 0.058 | 0.121 | -5.922 |  |  | 1 |
| 844 | 45.69 | 0 | g__UBA1191 | s__ | 0.029 | 0.001 | 0.003 | 5.822 | 1 |  |  |
| 401 | 47.24 | 1.68 | g__UBA1213 | s__ | 0.035 | 0.000 | 0.065 | 8.187 |  |  | 1 |
| 669 | 62.12 | 2.01 | g__UBA1213 | s__ | 0.097 | 0.000 | 0.051 | 8.960 |  |  | 1 |
| 805 | 12.07 | 0 | g__UBA1213 | s__ | 0.023 | 0.000 | 0.066 | 8.499 |  |  | 1 |
| 900 | 81.97 | 14.97 | g__UBA1213 | s__UBA1213 sp002351795 | 0.166 | 0.001 | 0.051 | 7.819 |  |  | 1 |
| 677 | 88.76 | 2.35 | g__UBA1213 | s__UBA1213 sp900322105 | 0.315 | 0.001 | 0.125 | 8.977 |  |  | 1 |
| 337 | 94.20 | 12.14 | g__UBA1777 | s__ | 0.486 | 0.010 | 0.002 | 5.668 | 1 |  |  |
| 798 | 85.66 | 48.43 | g__UBA1777 | s__ | 0.480 | 0.005 | 0.001 | 6.639 | 1 |  |  |
| 303 | 84.64 | 120.3 | g__UBA1777 | s__ | 0.400 | 0.003 | 0.003 | 7.177 | 1 |  |  |
| 594 | 66.02 | 30.34 | g__UBA1777 | s__ | 0.274 | 0.002 | 0.001 | 6.788 | 1 |  |  |
| 735 | 57.99 | 27.51 | g__UBA1777 | s__ | 0.274 | 0.002 | 0.002 | 6.859 | 1 |  |  |
| 317 | 86.29 | 147.18 | g__UBA1777 | s__ | 0.212 | 0.009 | 0.001 | 4.563 | 1 |  |  |
| 66 | 31.38 | 0 | g__UBA1777 | s__ | 0.058 | 0.000 | 0.000 | 7.448 | 1 |  |  |
| 151 | 18.10 | 3.45 | g__UBA1777 | s__ | 0.039 | 0.000 | 0.004 | 6.961 | 1 |  |  |
| 576 | 18.97 | 3.45 | g__UBA1777 | s__ | 0.031 | 0.000 | 0.018 | 6.608 | 1 |  |  |
| 61 | 25.86 | 3.45 | g__UBA1777 | s__ | 0.014 | 0.000 | 0.001 | 7.827 | 1 |  |  |
| 903 | 31.03 | 0 | g__UBA1777 | s__ | 0.014 | 0.000 | 0.000 | 6.039 | 1 |  |  |
| 728 | 14.66 | 0 | g__UBA1777 | s__ | 0.010 | 0.000 | 0.000 | 6.789 | 1 |  |  |
| 771 | 15.52 | 0 | g__UBA1777 | s__UBA1777 sp002371675 | 0.030 | 0.000 | 0.004 | 6.444 | 1 |  |  |
| 172 | 55.85 | 0.34 | g__UBA1777 | s__UBA1777 sp003150355 | 0.008 | 0.046 | 0.002 | -2.599 |  | 1 |  |
| 481 | 86.98 | 5.03 | g__UBA1777 | s__UBA1777 sp900317375 | 0.690 | 0.002 | 0.000 | 8.171 | 1 |  |  |
| 710 | 42.63 | 3.51 | g__UBA2727 | s__UBA2727 sp900315505 | 0.007 | 0.029 | 0.007 | -2.088 |  | 1 |  |
| 385 | 98.25 | 5.45 | g__UBA2727 | s__UBA2727 sp900316165 | 0.060 | 0.237 | 0.029 | -1.982 |  | 1 |  |
| 577 | 15.79 | 0 | g__UBA2727 | s__UBA2727 sp900317825 | 0.005 | 0.019 | 0.001 | -1.907 |  | 1 |  |
| 19 | 51.86 | 0.67 | g__UBA2821 | s__ | 0.015 | 0.009 | 0.643 | 0.728 |  |  | 1 |
| 45 | 22.15 | 0.63 | g__UBA2856 | s__ | 0.025 | 0.084 | 0.016 | -1.731 |  | 1 |  |
| 714 | 91.87 | 17.77 | g__UBA2856 | s__ | 0.551 | 0.006 | 0.010 | 6.536 | 1 |  |  |
| 355 | 85.96 | 68.37 | g__UBA2856 | s__ | 0.271 | 0.011 | 0.000 | 4.669 | 1 |  |  |
| 703 | 92.98 | 55.02 | g__UBA2856 | s__ | 0.247 | 0.004 | 0.003 | 5.790 | 1 |  |  |
| 656 | 80.26 | 2.69 | g__UBA2856 | s__ | 0.211 | 0.002 | 0.000 | 6.653 | 1 |  |  |
| 20 | 35.96 | 1.75 | g__UBA2856 | s__ | 0.197 | 0.003 | 0.005 | 5.994 | 1 |  |  |
| 334 | 75.90 | 13.4 | g__UBA2856 | s__ | 0.167 | 0.002 | 0.013 | 6.585 | 1 |  |  |
| 395 | 60.34 | 15.59 | g__UBA2856 | s__ | 0.147 | 0.001 | 0.006 | 8.036 | 1 |  |  |
| 81 | 57.10 | 3.83 | g__UBA2856 | s__ | 0.103 | 0.002 | 0.006 | 6.032 | 1 |  |  |
| 269 | 48.25 | 1.75 | g__UBA2856 | s__ | 0.054 | 0.004 | 0.044 | 3.778 | 1 |  |  |
| 35 | 71.45 | 6.03 | g__UBA2856 | s__UBA2856 sp002371345 | 0.171 | 0.002 | 0.004 | 6.767 | 1 |  |  |
| 485 | 80.70 | 3.51 | g__UBA2856 | s__UBA2856 sp900318615 | 0.063 | 0.232 | 0.058 | -1.886 |  |  | 1 |
| 428 | 94.30 | 2.23 | g__UBA2856 | s__UBA2856 sp900319065 | 0.056 | 1.482 | 0.061 | -4.732 |  |  | 1 |
| 861 | 61.05 | 34.01 | g__UBA2862 | s__ | 0.324 | 0.003 | 0.003 | 6.785 | 1 |  |  |
| 386 | 87.30 | 75.71 | g__UBA2862 | s__ | 0.148 | 0.002 | 0.001 | 5.964 | 1 |  |  |
| 884 | 59.62 | 7.12 | g__UBA2862 | s__ | 0.085 | 0.025 | 0.431 | 1.783 |  |  | 1 |
| 88 | 17.54 | 0 | g__UBA2912 | s__ | 0.025 | 0.238 | 0.002 | -3.252 |  | 1 |  |
| 676 | 49.12 | 0 | g__UBA2912 | s__UBA2912 sp900314775 | 0.003 | 0.109 | 0.175 | -5.247 |  |  | 1 |
| 777 | 53.62 | 0 | g__UBA2912 | s__UBA2912 sp900317095 | 0.007 | 0.041 | 0.000 | -2.601 |  | 1 |  |
| 719 | 96.14 | 8.2 | g__UBA2922 | s__ | 0.751 | 0.005 | 0.000 | 7.379 | 1 |  |  |
| 641 | 65.48 | 18.94 | g__UBA2943 | s__ | 0.140 | 0.001 | 0.000 | 6.813 | 1 |  |  |
| 155 | 22.41 | 1.72 | g__UBA3212 | s__ | 0.030 | 0.002 | 0.069 | 4.073 |  |  | 1 |
| 886 | 82.55 | 1.34 | g__UBA3212 | s__UBA3212 sp002363615 | 0.071 | 0.002 | 0.099 | 5.536 |  |  | 1 |
| 14 | 19.83 | 1.72 | g__UBA3305 | s__ | 0.019 | 0.000 | 0.000 | 5.846 | 1 |  |  |
| 493 | 97.22 | 1.65 | g__UBA3738 | s__ | 0.088 | 0.002 | 0.000 | 5.775 | 1 |  |  |
| 39 | 49.89 | 2.91 | g__UBA3738 | s__ | 0.038 | 0.000 | 0.000 | 6.679 | 1 |  |  |
| 842 | 41.56 | 2.29 | g__UBA3766 | s__ | 0.025 | 0.000 | 0.001 | 6.680 | 1 |  |  |
| 622 | 41.29 | 2.84 | g__UBA3766 | s__ | 0.018 | 0.000 | 0.007 | 6.238 | 1 |  |  |
| 293 | 87.86 | 16.62 | g__UBA3855 | s__ | 0.037 | 0.000 | 0.074 | 7.118 |  |  | 1 |
| 588 | 90.60 | 65.94 | g__UBA3855 | s__ | 0.233 | 0.001 | 0.068 | 8.473 |  |  | 1 |
| 787 | 54.28 | 3.83 | g__UBA3855 | s__ | 0.057 | 0.000 | 0.000 | 7.207 | 1 |  |  |
| 625 | 41.38 | 5.17 | g__UBA3855 | s__ | 0.035 | 0.000 | 0.158 | 6.640 |  |  | 1 |
| 709 | 25.00 | 0 | g__UBA3855 | s__ | 0.022 | 0.000 | 0.069 | 7.466 |  |  | 1 |
| 476 | 18.55 | 1.1 | g__UBA3855 | s__ | 0.036 | 0.001 | 0.001 | 5.572 | 1 |  |  |
| 448 | 45.67 | 4.77 | g__UBA4246 | s__UBA4246 sp002391225 | 0.119 | 0.001 | 0.001 | 6.567 | 1 |  |  |
| 891 | 66.43 | 12.25 | g__UBA4263 | s__ | 0.224 | 0.011 | 0.001 | 4.286 | 1 |  |  |
| 150 | 16.47 | 0 | g__UBA4263 | s__ | 0.018 | 0.000 | 0.001 | 6.217 | 1 |  |  |
| 26 | 93.29 | 0.41 | g__UBA4285 | s__ | 0.013 | 0.109 | 0.134 | -3.100 |  |  | 1 |
| 56 | 32.89 | 2.01 | g__UBA4285 | s__ | 0.003 | 0.151 | 0.078 | -5.847 |  |  | 1 |
| 875 | 50.88 | 0 | g__UBA4285 | s__ | 0.003 | 0.091 | 0.088 | -4.777 |  |  | 1 |
| 569 | 45.61 | 0 | g__UBA4285 | s__UBA4285 sp900314255 | 0.002 | 0.087 | 0.001 | -5.738 |  | 1 |  |
| 239 | 92.75 | 0.48 | g__UBA629 | s__UBA629 sp900316625 | 0.136 | 0.235 | 0.089 | -0.783 |  |  | 1 |
| 36 | 57.93 | 0.97 | g__UBA629 | s__UBA629 sp900316665 | 0.008 | 0.330 | 0.280 | -5.349 |  |  | 1 |
| 463 | 64.11 | 0.97 | g__UBA629 | s__UBA629 sp900317915 | 0.050 | 0.106 | 0.004 | -1.074 |  | 1 |  |
| 193 | 36.84 | 0 | g__UBA629 | s__UBA629 sp900317915 | 0.008 | 0.019 | 0.010 | -1.282 |  | 1 |  |
| 244 | 38.27 | 0 | g__UBA9722 | s__UBA9722 sp900315015 | 0.039 | 0.046 | 0.312 | -0.245 |  |  | 1 |
| 607 | 83.89 | 3.97 | g__ | s__ | 0.120 | 0.166 | 0.091 | -0.468 |  |  | 1 |
| 918 | 98.78 | 0.95 | g__Acidaminococcus | s__Acidaminococcus fermentans | 0.025 | 0.376 | 0.056 | -3.933 |  |  | 1 |
| 665 | 84.63 | 0.11 | g__Acidaminococcus | s__Acidaminococcus sp900315205 | 0.082 | 0.162 | 0.028 | -0.979 |  | 1 |  |
| 350 | 18.10 | 0 | g__Anaerovibrio | s__Anaerovibrio lipolyticus_A | 0.003 | 0.000 | 0.020 | 4.618 | 1 |  |  |
| 18 | 99.82 | 0.63 | g__Dialister | s__Dialister sp002471975 | 0.023 | 2.310 | 0.005 | -6.654 |  | 1 |  |
| 432 | 31.19 | 1.72 | g__Dialister | s__Dialister sp900314595 | 0.052 | 0.320 | 0.150 | -2.633 |  |  | 1 |
| 753 | 15.52 | 0 | g__Megasphaera | s__Megasphaera elsdenii | 0.002 | 0.010 | 0.116 | -2.300 |  |  | 1 |
| 205 | 14.04 | 0 | g__Selenomonas_A | s__ | 0.010 | 0.001 | 0.078 | 3.617 |  |  | 1 |
| 12 | 31.58 | 0 | g__Succiniclasticum | s__ | 0.128 | 0.005 | 0.000 | 4.542 | 1 |  |  |
| 730 | 78.64 | 5.49 | g__Succiniclasticum | s__Succiniclasticum sp900315345 | 0.137 | 0.081 | 0.801 | 0.766 |  |  | 1 |
| 747 | 81.94 | 10.49 | g__Succiniclasticum | s__Succiniclasticum sp900315925 | 0.807 | 0.032 | 0.000 | 4.670 | 1 |  |  |
| 95 | 12.28 | 0 | g__ | s__ | 0.019 | 0.006 | 0.493 | 1.756 |  |  | 1 |
| 118 | 51.68 | 6.41 | g__ | s__ | 0.004 | 0.000 | 0.090 | 3.480 |  |  | 1 |
| 137 | 64.67 | 1.71 | g__ | s__ | 0.008 | 0.085 | 0.281 | -3.402 |  |  | 1 |
| 310 | 42.19 | 0 | g__ | s__ | 0.000 | 0.006 | 0.156 | -5.657 |  |  | 1 |
| 552 | 81.58 | 0 | g__ | s__ | 0.015 | 0.041 | 0.117 | -1.478 |  |  | 1 |
| 732 | 19.12 | 10.34 | g__ | s__ | 0.011 | 0.000 | 0.001 | 6.036 | 1 |  |  |
| 169 | 69.12 | 13.79 | g__HOT-345 | s__ | 0.026 | 0.000 | 0.006 | 8.175 | 1 |  |  |
| 454 | 55.02 | 6.9 | g__HOT-345 | s__ | 0.033 | 0.001 | 0.001 | 5.463 | 1 |  |  |
| 91 | 56.98 | 2.71 | g__UBA2834 | s__ | 0.036 | 0.066 | 0.142 | -0.872 |  |  | 1 |
| 144 | 28.14 | 0 | g__UBA2834 | s__ | 0.007 | 0.000 | 0.038 | 6.112 | 1 |  |  |
| 224 | 27.59 | 7.05 | g__UBA2834 | s__ | 0.005 | 0.000 | 0.048 | 5.437 | 1 |  |  |
| 924 | 63.45 | 8.62 | g__UBA2834 | s__ | 0.019 | 0.017 | 0.602 | 0.127 |  |  | 1 |
| 46 | 36.31 | 0 | g__UBA2834 | s__UBA2834 sp002371895 | 0.009 | 0.002 | 0.382 | 2.188 |  |  | 1 |
| 889 | 22.33 | 5.42 | g__UBA3291 | s__ | 0.004 | 0.000 | 0.012 | 8.510 | 1 |  |  |
| 883 | 78.40 | 2.3 | g__UBA2804 | s__UBA2804 sp900319705 | 0.586 | 0.003 | 0.014 | 7.567 | 1 |  |  |
| 178 | 43.34 | 13.01 | g__UBA2810 | s__ | 2.629 | 3.939 | 0.012 | -0.583 |  | 1 |  |
| 688 | 12.54 | 0 | g__UBA2810 | s__ | 0.503 | 0.252 | 0.654 | 0.999 |  |  | 1 |
| 223 | 64.73 | 9.12 | g__UBA2810 | s__UBA2810 sp900317945 | 4.498 | 7.249 | 0.011 | -0.689 |  | 1 |  |
| 190 | 23.04 | 0 | g__UBA3636 | s__ | 0.090 | 0.000 | 0.009 | 8.638 | 1 |  |  |
| 32 | 10.34 | 0 | NA | NA | 0.008 | 0.000 | 0.015 | 4.426 | 1 |  |  |
| 58 | 10.23 | 0 | NA | NA | 0.009 | 0.000 | 0.017 | 6.039 | 1 |  |  |
| 71 | 12.28 | 0 | NA | NA | 0.014 | 0.001 | 0.000 | 4.665 | 1 |  |  |
| 79 | 15.67 | 0 | NA | NA | 0.009 | 0.000 | 0.018 | 7.252 | 1 |  |  |
| 109 | 11.21 | 0 | NA | NA | 0.003 | 0.003 | 0.365 | 0.278 |  |  | 1 |
| 129 | 12.52 | 0 | NA | NA | 0.005 | 0.032 | 0.001 | -2.781 |  | 1 |  |
| 160 | 10.53 | 3.51 | NA | NA | 0.005 | 0.000 | 0.001 | 3.845 | 1 |  |  |
| 166 | 11.21 | 0 | NA | NA | 0.004 | 0.000 | 0.001 | 5.286 | 1 |  |  |
| 186 | 13.79 | 0 | NA | NA | 0.021 | 0.000 | 0.044 | 8.595 | 1 |  |  |
| 259 | 12.07 | 0 | NA | NA | 0.096 | 0.005 | 0.003 | 4.123 | 1 |  |  |
| 271 | 14.04 | 1.75 | NA | NA | 0.059 | 0.001 | 0.000 | 5.955 | 1 |  |  |
| 309 | 10.34 | 0 | NA | NA | 0.005 | 0.000 | 0.019 | 6.362 | 1 |  |  |
| 331 | 10.34 | 0 | NA | NA | 0.093 | 0.002 | 0.000 | 5.285 | 1 |  |  |
| 379 | 11.21 | 0 | NA | NA | 0.011 | 0.000 | 0.000 | 6.467 | 1 |  |  |
| 486 | 10.34 | 0 | NA | NA | 0.020 | 0.003 | 0.027 | 2.867 | 1 |  |  |
| 507 | 10.53 | 0 | NA | NA | 0.009 | 0.000 | 0.008 | 7.192 | 1 |  |  |
| 524 | 10.34 | 0 | NA | NA | 0.013 | 0.000 | 0.001 | 5.354 | 1 |  |  |
| 536 | 13.79 | 15.52 | NA | NA | 0.048 | 0.107 | 0.061 | -1.142 |  |  | 1 |
| 560 | 17.24 | 0 | NA | NA | 0.053 | 0.001 | 0.000 | 6.577 | 1 |  |  |
| 612 | 15.52 | 0 | NA | NA | 0.003 | 0.001 | 0.943 | 1.249 |  |  | 1 |
| 624 | 13.79 | 0 | NA | NA | 0.005 | 0.000 | 0.036 | 7.423 | 1 |  |  |
| 642 | 10.53 | 0 | NA | NA | 0.015 | 0.000 | 0.034 | 6.927 | 1 |  |  |
| 664 | 13.79 | 0 | NA | NA | 0.001 | 0.001 | 0.034 | 0.130 | 1 |  |  |
| 716 | 10.64 | 0 | NA | NA | 0.000 | 0.005 | 0.000 | -5.287 |  | 1 |  |
| 772 | 10.34 | 0 | NA | NA | 0.022 | 0.001 | 0.012 | 4.263 | 1 |  |  |
| 792 | 10.34 | 0 | NA | NA | 0.064 | 0.000 | 0.000 | 8.018 | 1 |  |  |
| 828 | 15.52 | 0 | NA | NA | 0.003 | 0.012 | 0.027 | -1.762 |  | 1 |  |
| 829 | 10.71 | 0 | NA | NA | 0.034 | 0.000 | 0.000 | 6.580 | 1 |  |  |
| 863 | 10.27 | 1.72 | NA | NA | 0.024 | 0.022 | 0.330 | 0.170 |  |  | 1 |
| **Total** |  |  |  |  |  |  |  |  | **274** | **89** | **198** |
